# Simultaneously Measuring Image Features and Resolution in Live-Cell STED Images

**DOI:** 10.1016/j.bpj.2018.07.028

**Published:** 2018-08-04

**Authors:** Andrew E.S. Barentine, Lena K. Schroeder, Michael Graff, David Baddeley, Joerg Bewersdorf

**Affiliations:** 1 Department of Cell Biology, Yale University School of Medicine, New Haven, Connecticut; 2 Department of Biomedical Engineering, Yale University, New Haven, Connecticut; 3 Auckland Bioengineering Institute, University of Auckland, Auckland, New Zealand

## Abstract

Reliable interpretation and quantification of cellular features in fluorescence microscopy requires an accurate estimate of microscope resolution. This is typically obtained by measuring the image of a nonbiological proxy for a point-like object, such as a fluorescent bead. Although appropriate for confocal microscopy, bead-based measurements are problematic for stimulated emission depletion microscopy and similar techniques where the resolution depends critically on the choice of fluorophore and acquisition parameters. In this article, we demonstrate that for a known geometry (e.g., tubules), the resolution can be measured in situ by fitting a model that accounts for both the point spread function (PSF) and the fluorophore distribution. To address the problem of coupling between tubule diameter and PSF width, we developed a technique called nested-loop ensemble PSF fitting. This approach enables extraction of the size of cellular features and the PSF width in fixed-cell and live-cell images without relying on beads or precalibration. Nested-loop ensemble PSF fitting accurately recapitulates microtubule diameter from stimulated emission depletion images and can measure the diameter of endoplasmic reticulum tubules in live COS-7 cells. Our algorithm has been implemented as a plugin for the PYthon Microscopy Environment, a freely available and open-source software.

## Introduction

Fluorescence microscopy images never represent the underlying object perfectly, failing to discern details smaller than a certain size. This imperfection is described by the system’s point-spread function (PSF). Knowledge of the PSF is essential when interpreting the images produced and in ensuring that quantitative measurements are accurate. For many purposes, it is sufficient to summarize the effects of the PSF in a simple resolution metric (e.g., the full-width at half maximum (FWHM)). A popular method for obtaining the PSF FWHM is extracting an intensity line profile from a fluorescent bead image and either directly measuring the FWHM or estimating it more accurately by fitting a Gaussian or Lorentzian (in the case of stimulated emission depletion (STED)) model to the profile. For diffraction-limited microscopes, beads can be regarded as point-sources because they are significantly smaller than the FWHM of the PSF (beads are typically 20–100 nm compared to the ∼250 nm PSF FWHM), and the fit FWHM is taken to be that of the PSF.

When considering STED microscopy, where the PSF FWHM is typically 25–70 nm, the assumption that the PSF is much larger than the bead size is no longer valid, as beads whose size is similar to that of the PSF are often needed to achieve reasonable signal levels. The resolution in STED microscopy is also strongly affected by laser powers, sample-induced aberrations, and the depletion cross-section of the dye, which is additionally dependent on the local environment, making beads a poor proxy for the true resolution achieved when imaging cellular samples.

Measuring STED resolution on a target in the same cellular environment labeled with the same dye(s) and imaged with the same laser powers avoids these issues. Microtubules labeled with the same fluorescent dye as the final target structure are an attractive candidate that can be readily prepared. The simplest and most common labeling protocol that usually results in bright stainings is indirect immunofluorescence. Labeling the 25-nm outer diameter of a microtubule with primary and secondary antibodies results in a structure that is 60 nm in diameter as observed using electron microscopy (1). However, this is within the size range of a STED PSF, so as with beads the thickness of the structure is nonnegligible when quantifying the resolution of a microtubule image. To determine the impact finite object size has on resolution quantification using the popular Gaussian- and Lorentzian-fitting techniques, we simulated intensity line profiles perpendicular to the long axis of antibody-labeled microtubules imaged at various resolutions and fit them with Gaussian and Lorentzian functions.

**Figure 1:**
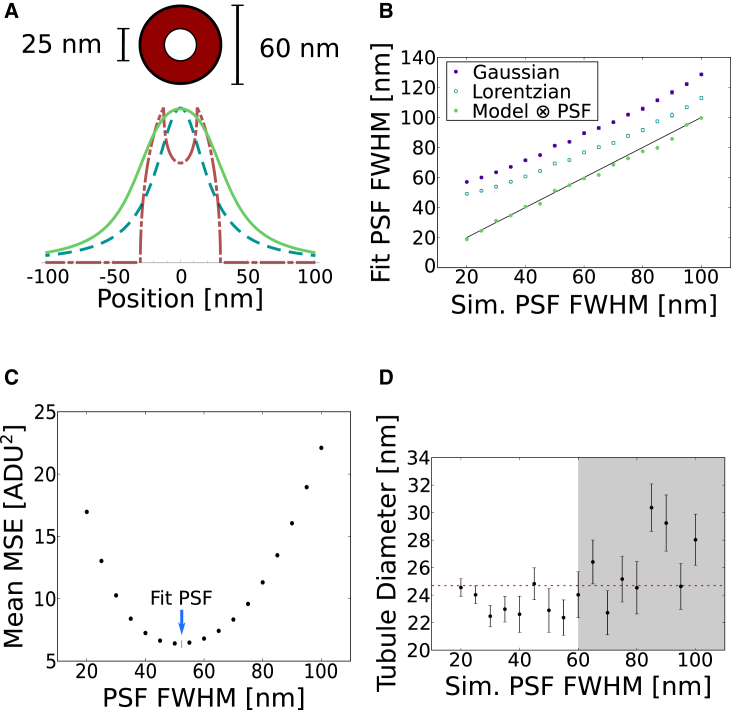
(*A*) Annulus used to model fluorophore location, where antibodies and fluorophores are bound to the surface of a 25-nm diameter microtubule (1). The red dash-dot curve represents a projection of the fluorophore distribution (summing over the axial dimension), the teal dashed curve a Lorentzian function that models the PSF, and the green solid curve the convolution of the other two. (*B*) Microtubule line-profiles were simulated at various resolutions using Lorentzian PSFs, with shot noise added before being fit with simple Gaussian (*purple*) and Lorentzian (*teal hollow*) functions. The same profiles were also fit using NEP fitting (*green*), which results in good agreement with the ground truth of the simulations (*black line*). N = 50 profiles were fit for each simulated PSF width. (*C*) Plot of mean MSE for fits performed with the Lorentzian-convolved model function at specified PSF widths on simulated microtubule profiles. These profiles were generated with a 50-nm PSF and added shot noise. NEP fitting minimizes the mean MSE with a PSF FWHM of 51.2 nm, as indicated by the blue arrow. (*D*) Plot of microtubule diameters determined by NEP fitting, where images were simulated at various resolutions (N = 50 profiles at each PSF width, error bars denote standard error of the mean). The ground truth diameter was 25 nm for all profiles, as shown by the dashed red line. The gray region of the plot indicates where the simulated PSF FWHM is larger than the antibody-coated tubule structure, which results in less accurate tubule diameter fits. To see this figure in color, go online.

We modeled the fluorophore distribution for the primary and secondary antibody labeled microtubule as an annulus of 25-nm inner diameter and 60-nm outer diameter, as measured for densely-labeled microtubules (1) (Fig. 1 *A*, *top*). For most STED microscopes, the axial (z) PSF FWHM is considerably larger (500–700 nm) than the FWHM along the lateral (xy) directions. This means that the entire cross-section of a microtubule is effectively summed along the axial dimension during imaging, producing the red dash-dot curve in Fig. 1 *A*. The imaging process is simulated by convolving the fluorophore distribution with the PSF model (Fig. 1 *A*, *teal dashed curve*). For these simulations, we modeled the PSF as a Lorentzian, a common STED PSF approximation (2, 3) that represents our experimental PSF better than a Gaussian (see Fig. S1 in the Supporting Material). The resulting cross section of a microtubule imaged with a 50-nm FWHM PSF is shown in Fig. 1 *A* (*green solid curve*).

We simulated line profiles across microtubules imaged with STED resolutions ranging from 20 to 100 nm (PSF FWHM) with added shot noise and fit them with simple Lorentzian and Gaussian functions. The FWHM of the Lorentzian fits, and in particular the Gaussian fits, were substantially larger than the PSF FWHM they were simulated with (Fig. 1 *B*), confirming that simple fitting of these models does not result in an accurate resolution measure. The accuracy deteriorates, as expected, at higher resolutions (smaller PSF FWHM), making it particularly problematic for STED microscopy, in which systematic errors of 100% can easily occur.

Similar Gaussian or Lorentzian fits in which the width is interpreted as the size of an imaged structure rather than resolution are popular in various types of fluorescence (super-resolution) microscopy. Interpreting Fig. 1 *B* this way shows that this approach only yields reasonable results when the PSF is much smaller than the imaged structure. We conclude that using the FWHM of Gaussian or Lorentzian fits is an unreliable measure for both resolution and feature size quantification.

As an alternative to fitting of line profiles, Fourier ring correlation, a powerful model-free method of measuring relative resolution in images of similar objects (4, 5), has been used to quantify STED resolution (6). Unfortunately, Fourier ring correlation results are highly dependent on the structure being imaged, subject to the same limitations relating to object dimensions as simple profile fits, and are not directly translatable into FWHM values.

In order to accurately determine microscope resolution or feature size from line profile cross-sections, both the microscope PSF and the geometric distribution of fluorophores on the labeled structure must be modeled in the function used for fitting. Previous efforts have held-fixed one of these parameters, assuming that either the PSF or the structure size is already known and enforcing this assumption either during fitting (7) or in a simulation to quantify the FWHM bias of the fit (8). However, these approaches are limited because in biological STED microscopy, both structure size and resolution are typically unknown. Fitting both of these parameters simultaneously, on the other hand, is difficult; increases in either parameter give rise to an increased profile width, albeit with subtly different effects on profile shape. At the signal-to-noise (SNR) level typical of a single profile, it is difficult to separate the effects of the two parameters. This coupling can result in inaccurate estimates for both values. Here, we present a tool that overcomes this challenge and allows simultaneous fitting of structure size and PSF width.

### Ensemble PSF Fitting

We enable robust simultaneous determination of PSF width and structure size by fitting multiple line profiles as an ensemble, exploiting prior knowledge that the PSF width should be the same for each profile. We accomplish this by performing a two-layer nested fit, such that in the inner fit, all tubules are fit with the same PSF FWHM, *γ*, and the mean squared error (MSE) for each tubule is reported. The MSE averaged over all tubule fits as a function of *γ* has a propensity to be well behaved and smooth (Fig. 1 *C*). The outer fit is then responsible for finding the value of *γ* which minimizes the mean MSE. This technique, which we refer to as nested-loop ensemble PSF (NEP) fitting, constrains the fit enough that accurate PSF widths and microtubule diameters can be determined, as shown in Fig. 1, *B* and *D*.

NEP fitting using the antibody-coated tubule model yielded significantly better results than fitting with plain Gaussian or Lorentzian functions, and the PSF widths calculated by the fit are in close agreement with the ground truth (Fig. 1 *B*). Simultaneously, it yielded accurate measures of the simulated microtubule diameter, 25 nm, for all simulated PSFs with FWHM equal to or less than 60 nm, which is the value at which the PSF FWHM becomes larger than the outer diameter of the antibody coat (Fig. 1 *D*). For structures whose size does not vary in a cell (e.g., microtubule diameters), the structure size can additionally be constrained as an ensemble parameter during the fit, although we did not find this to be a necessary step.

We carried out additional simulations to determine the robustness of NEP fitting on low SNR data as well as data obtained with aberrated PSFs. Not surprisingly, the variability in fit tubule diameters for individual profiles is increased at lower SNRs (Fig. S2), yet NEP fitting estimates for tubule diameter and PSF width do not suffer from systematic errors. Systematic changes in initial fit parameter estimates led to negligible differences in the fit outcomes (see Fig. S3), demonstrating the robustness of the nested fit approach. We then simulated STED PSFs with various aberrations and found that sizes of the convolved structures were still accurately determined (Fig. S4). Additionally, the PSF width estimates from NEP fitting were in good agreement with the FWHM of the simulated (aberrated) PSFs (Fig. S5).

### Software and Validation

**Figure 2:**
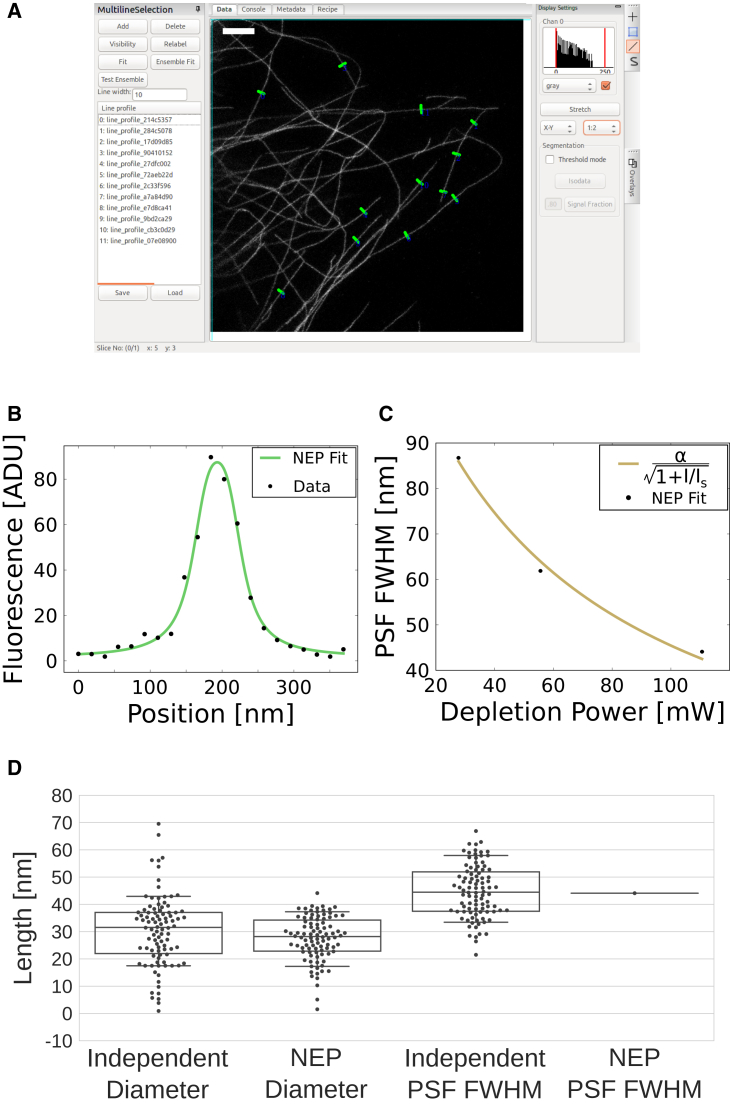
(*A*) PYME GUI showing a STED image of immunolabeled microtubules in a COS-7 cell, imaged with 111-mW STED laser power. Green lines show user-selected profiles to be fit. (*B*) Plot of raw data and NEP fit of a microtubule profile from (*A*) are shown. (*C*) Plot of NEP-fitted PSF widths from STED images of microtubules acquired with different STED powers, which scales as expected by theory (n = 74, n = 71, and n = 94 profiles extracted from N = 8, N = 8, and N = 12 images of N = 3, N = 3, and N = 6 cells, acquired at 28, 56, and 111-mW STED laser powers, respectively), are shown. (*D*) Swarm- and box-plots of microtubule diameters and PSF FWHM values determined using NEP fitting, where the PSF is constrained to be the same for all microtubule line profile cross sections, and without NEP fitting (standard least-squares fitting), where the PSF is varied independently for each tubule fit, are shown. To see this figure in color, go online.

We implemented NEP fitting for STED images of label-filled or surface-labeled cylindrical structures in the PYthon Microscopy Environment (PYME). Line profiles of a user-defined width are extracted from images loaded into PYME, after which they can be fit using a variety of model functions. Alternatively, they can be saved or appended to two file formats (Hierarchical Data Format, JavaScript Object Notation) for later analysis or ensemble fitting with profiles from multiple images. Fitting performed through the graphical user interface (GUI) saves results to an Hierarchical Data Format table and generates an HTML report (see Fig. S6 for an example report). The line profile extraction GUI is shown in Fig. 2 *A*.

STED PSF width is dependent on the STED laser power with a scaling of $\alpha /\sqrt{1+I/I_{s}}$, where *I* is the depletion intensity and *α* and *I*_*s*_ are constants (9). To test the efficacy of ensemble fitting on real data, we imaged primary and secondary antibody-labeled microtubules using a Leica SP8 STED 3X microscope (Leica Microsystems, Wetzlar, Germany) at different STED laser powers. Fig. 2 *C* shows that our fit is responsive to changes in PSF size and can reproduce the expected scaling of PSF width with respect to the STED laser power.

To determine if our NEP fitting approach in which the PSF width is constrained to a single, global value for all profiles is beneficial, we compared its results with those obtained when both PSF width and tubule diameter were optimized on a per-profile basis. We performed this comparison on images recorded with 111-mW depletion power. Although fits performed without an ensemble PSF have more degrees of freedom and therefore usually yield smaller residuals, they come at the expense of accuracy in the measured values. This can be seen in Fig. 2 *D*, where the standard least squares fit results in an average microtubule diameter of 30 ± 13 nm (mean ± standard deviation (SD)), compared to the NEP-fitted value of 28 ± 8 nm (mean ± SD). NEP fitting, with its global PSF constraint, improves the measurement of the microtubule diameter, as evident from the reduced spread in tubule diameters and average value closer to the expected 25 nm. The PSF FWHM of standard least squares fitting was 45 ± 10 nm (mean ± SD), compared to the NEP-fitted value of 44 nm.

We note that fitting a single line profile would not provide a reliable measure of either PSF FWHM or tubule diameter and could lead to relative errors of 100% for both values. The minimum number of profiles necessary for robust NEP fitting depends on the fluorophore distribution and the relative PSF size. However, even for cases of low SNR, we found 100 profiles to be sufficient for the fluorophore distributions tested (see Fig. S2). To improve performance in low SNR images, we recommend averaging the line profiles over several pixels width during extraction (as can be done by entering, for example, 10 pixels for the “line width” in the GUI as described in the Supporting Material). This measure, which averages signal along a segment of the tubule, is also effective at reducing any discrepancies between the modeled and true fluorophore distribution that might occur as a result of sparse labeling.

### Application to live-cell images

**Figure 3:**
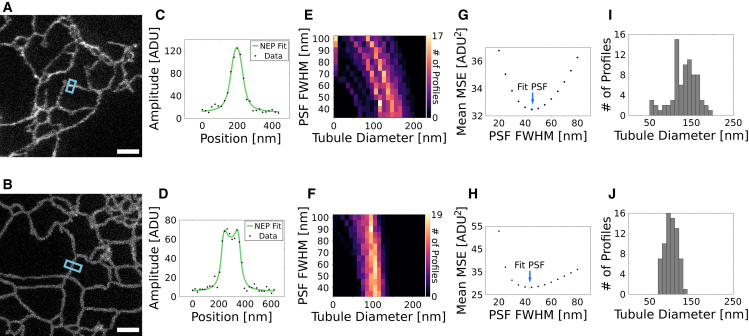
(A and B) Live-cell STED images of label-filled ((A) SNAP-KDEL) and membrane-labeled ((B) SNAP-Sec61*β*) ER. (C and D) Fluorescence line profiles, averaged over 10 pixels along the long axis of the tubule, extracted from (A) and (B), respectively, and fit using NEP fitting. (E and F) Heatmaps showing the coupling between tubule diameter and PSF FWHM for label-filled (E) and membrane-labeled (F) ER when standard least-squares fitting is performed with systematically varied PSF FWHM. n = 77 and n = 69 profiles were extracted from N = 7 and N = 7 STED images of N = 4 and N = 2 cells for (E) and (F), respectively. (G and H) Mean MSE values for fits shown in (E) and (F), respectively. The blue arrow indicates the PSF FWHM found by performing NEP fitting on the same tubule line profiles. (I and J) Label-filled (I) and membrane-labeled (J) ER tubule diameters fit using NEP fitting (45.8-nm PSF FWHM for SNAP-KDEL, 43.7-nm PSF FWHM for SNAP-Sec61*β*). The mean and standard deviations were 132 ± 30 and 101 ± 15 nm for (I) and (J), respectively. Scale bars, 1 *μ*m. ADU, analog-digital units. To see this figure in color, go online.

Although the robust PSF measurement in fixed cells by NEP fitting is a substantial improvement over bead calibrations, a large advantage of NEP fitting is that it can be performed on live-cell data for in situ resolution calibration in the most biologically relevant state. We applied ensemble PSF fitting in live-cell STED images of label-filled or membrane-labeled endoplasmic reticulum (ER) tubules using SNAP-KDEL or SNAP-Sec61*β*, respectively (Fig. 3, *A*–*D*). In order to fit the label-filled tubules, we modeled the fluorophore distribution perpendicular to the long axis of the tubule as a filled circle, which projects as $2\sqrt{R^{2}-x^{2}}$, where *R* is the radius. We then convolved this tubule profile with a Lorentzian to account for the imaging process. For membrane-labeled tubules, we modeled the fluorophore distribution as an annulus, like the antibody-labeled microtubules only with a thinner coat. SNAP-tag (10) is ∼4 nm in diameter, and the organic dye itself can be estimated to have a radius of 0.5 nm, assuming they are both globular (11), resulting in a 4.5-nm thick annulus. Fits of ER tubule diameter for various test PSF widths show stronger coupling between the tubule diameter and PSF width for label-filled tubules than membrane-labeled tubules (Fig. 3, *E* and *F*). The PSF width results from the NEP fits were, however, very similar for label-filled and membrane-labeled profiles (45.8 and 43.7 nm FWHM, respectively) as shown in Fig. 3, *G* and *H*.

Notably, the SD for both the label-filled and membrane-labeled ER tubule diameters is fairly large: 30 and 15 nm, respectively (with mean values of 132 and 101 nm). To test whether this variability in tubule diameter is primarily biological in nature or dominated by the SNR-limited fit precision, we simulated tubule profiles of known diameter with similar SNRs, convolved with 50-nm FWHM Lorentzians. The distributions of fitted diameters were narrower than those observed in the live-cell images, with SDs of only 5 nm for the membrane-labeled tubules and 12 nm for the label-filled tubules (Fig. S7). The larger range of label-filled tubule diameters is expected because the fluorophore distribution orthogonal to the long axis of the tubule looks more similar to the PSF than a surface-labeled fluorophore distribution. This is reflected in the live-cell data, where the tubule diameter is more strongly coupled to the PSF width for the label-filled tubules, as shown in the heat-maps of tubule diameter histograms when fit with various fixed PSF widths (Fig. 3, *E* and *F*). Since the standard deviations add in quadrature, we expect roughly 80% of the spread in tubule diameter to be biological in origin.

### Discussion

Traditional methods of resolution calibration in STED microscopy are problematic for biological quantification. The NEP fitting method introduced in this article provides a robust and practical alternative to both quantify the performance of a microscope as well as improve feature measurements within the image. Its implementation in a freely downloadable, open source, cross-platform software package allows for rapid adoption by others without requiring mathematical or programming expertise. The principle of ensemble fitting can be readily extended to other fluorescence microscopy modalities (e.g., confocal) by substituting a different functional representation of the PSF when producing the model function for fitting; the only requirement is that the labeling geometry of the structure is known. This known geometry is not limited to tubules and can be extended to fit objects like beads or vesicles, which would be useful for cell-trafficking studies. The accurate measure of PSF width afforded by NEP fitting can be used to quantify microscope performance under various conditions, refine models of organelle morphology, and remove uncertainty in parameter selection for deconvolution or other image enhancement algorithms.

### Software availability

All line profiles were drawn, extracted, and fit using the open source PYME and the NEP fitting plug-in, which are both freely available (12, 13). Supplemental data are provided alongside this article for readers to test the program.

## Author Contributions

A.E.S.B., D.B., and J.B. designed the research. A.E.S.B. developed the technique and model functions and performed simulations and data analysis. A.E.S.B., L.K.S., M.G., D.B., and J.B. wrote the manuscript. L.K.S. optimized and performed all imaging and live-cell sample preparations. A.E.S.B., M.G., and D.B. developed the plug-in for PYME. PYME has been developed by D.B.

## Supporting Citations

References (14, 15, 16, 17, 18, 19) appear in the Supporting Material.

## Supporting Material

Data S1. Supporting Data

Document S2. Article plus Supporting Material
