# Supplementary material for: Simultaneously Measuring Image Features and Resolution in Live-Cell STED Images
Source: Biophys J. 2018 Aug 4;115(6):951–6. doi: 10.1016/j.bpj.2018.07.028 (PMC6139878; doi:10.1016/j.bpj.2018.07.028)
Supplement: Document S1. Supporting Materials and Methods and Figs. S1–S7 [file mmc1.pdf]

Biophysical Journal, Volume *115*

Supplemental Information

Simultaneously Measuring Image Features and Resolution in Live-Cell STED Images

Andrew E.S. Barentine, Lena K. Schroeder, Michael Graff, David Baddeley, and Joerg Bewersdorf

Supplementary Figures

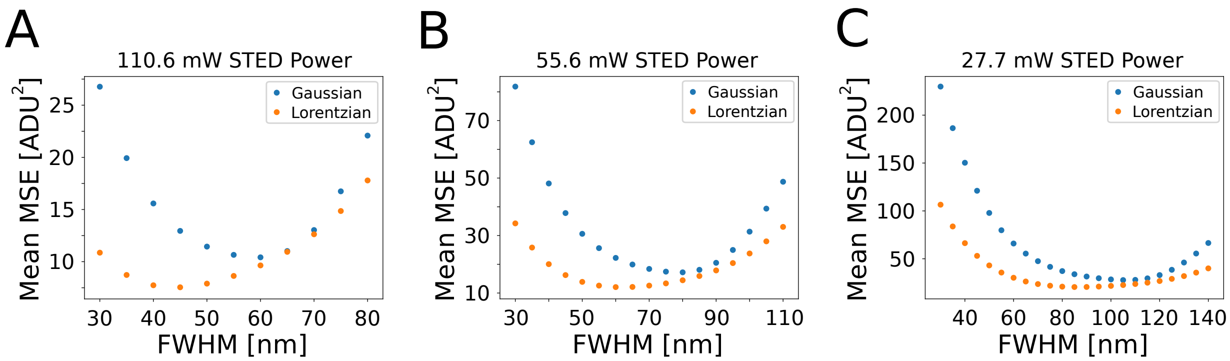

Figure S1: Comparison of Lorentzian and Gaussian PSF models applied to the microtubule data shown in figure 2. (**A**, **B**, **C**) MSE averaged over all profiles of NEP fit as a function of PSF FWHM when the NEP fitting model function is derived for a Gaussian (blue) or Lorentzian (orange) PSF model. STED laser powers were 110.6 mW, 55.6 mW, and 27.7 mW for (A), (B) and (C), respectively. The Lorentzian is a better model for our PSF as shown by the average MSE being consistently smaller compared to the Gaussian model for all tested STED laser powers. The difference is most notable (about a factor of 2) for 110.6 mW, which matches that used in our live-cell imaging. Approximating the depletion donut as a parabola with pulsed excitation, pulsed depletion and time-gated detection should produce a Gaussian-shaped effective PSF (1). Our experimental observation of a more Lorentzian-shaped effective PSF might be explained by the preferential excitation and depletion of certain dipole orientations of the fluorophore, which can have a significant impact on PSF shape.

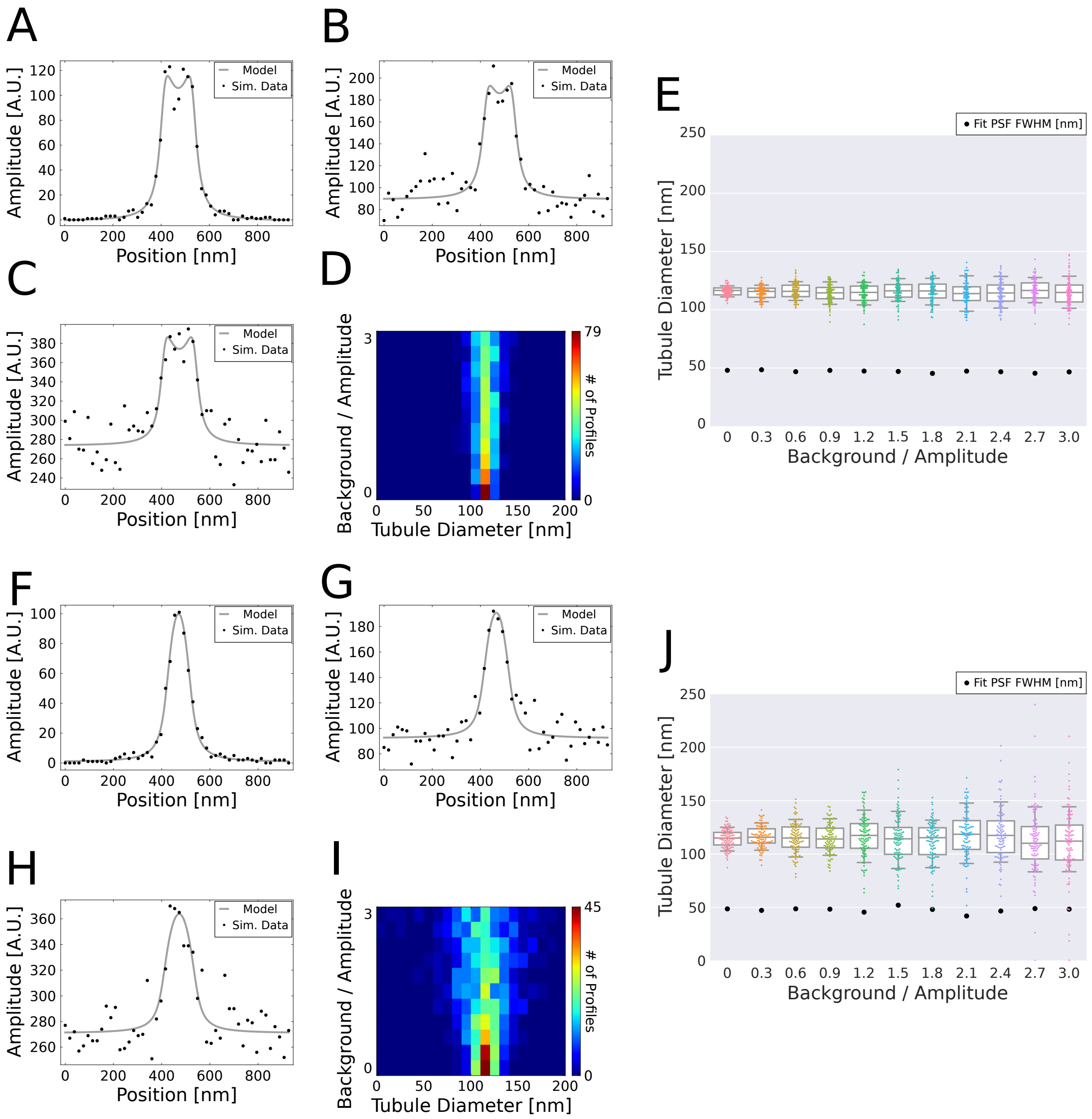

Figure S2: Effect of noise on ER tubule diameter and PSF fitting. (**A**, **B**, **C**) Simulated surface-labeled ER tubules with background to amplitude ratios of 0 (A), 0.9 (B), and 2.7 (C). (**D**) Heatmap of fitted diameters from NEP fits of simulated surface-labeled ER tubule profiles with varied background to amplitude ratios, and therefore varied signal-to-noise ratios. (**E**) Fitted diameters from NEP fits of simulated surface-labeled ER tubules with different background to amplitude ratios. The NEP-fit PSF width is plotted in black. N=100 simulated tubules for each ratio. (**F**, **G**, **H**) Simulated label-filled ER tubules with background to amplitude ratios of 0 (F), 0.9 (G), and 2.7 (H). (**I**) Heatmap of fitted diameters from NEP fits of simulated profiles of label-filled ER tubules with varied background to amplitude ratios, and therefore signal-to-noise ratios. (**J**) Fitted diameters from NEP fits of simulated label-filled ER tubules with different background to amplitude ratios. The NEP-fit PSF width is also plotted in black. N=100 simulated tubules for each ratio. The whiskers of boxplots represent the 10th and 90th percentiles of each distribution, the colored boxes cover the interquartile range, and the center line in each box denotes the median.

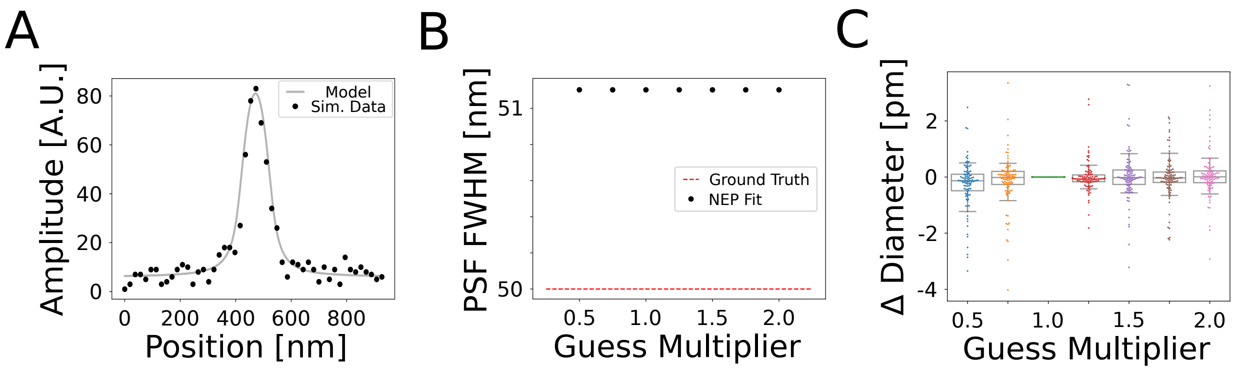

Figure S3: Both estimated tubule diameters and PSF FWHMs are independant of initial parameter guesses. (**A**) Example of a simulated 115 nm-diameter label-filled ER tubule convolved with a 50 nm FWHM Lorentzian PSF, with added Poisson noise. (**B**) NEP-fit PSF FWHM as a function of initial guesses for the fit parameters (including the PSF width). The initial guesses for fit parameters are calculated automatically, and then multiplied by a guess multiplier (abscissa) with the exception of the center position guess, which was shifted by one PSF FWHM (50 nm). The NEP-fit PSF width is plotted in black, the ground truth is represented by the dashed red line. While there is a small systematic error in the 1-nm range, the NEP-fit PSF width results are independent of the initial guesses for fit parameters. (**C**) The NEP-fit tubule diameters resulting from a guess multiplier of 1 (i.e. default automated guesses with the center position shifted by 50 nm) were subtracted from the NEP-fit tubule diameters resulting from the altered initial guesses. Notably, the effect of altering the initial guesses by a factor of two in either direction is very small, resulting in changes on the picometer-range, while the standard deviation of tubule diameters is roughly 10,000 times larger at 12.26 nm. N=100 simulated tubules for each guess multiplier. The whiskers of boxplots in (*B*) and (*C*) represent the 10th and 90th percentiles of each distribution, the boxes cover the interquartile range, and the center line in each box denotes the median.

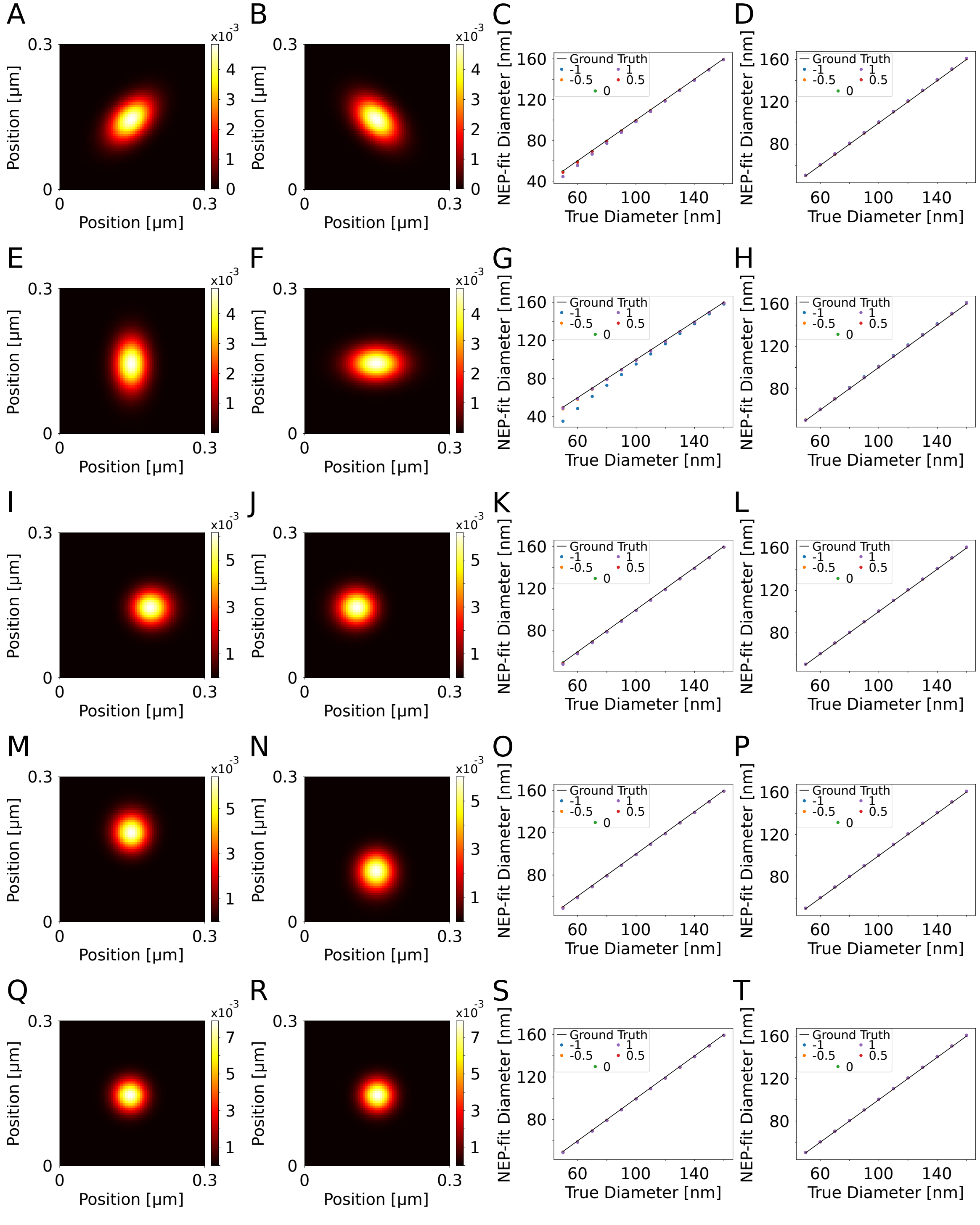

Figure S4: NEP-fitted tubule diameter measurements are robust to ±1 radian Zernike aberrations. A Gaussian (rather than Lorentzian) NEP-fitting model was used as the simulated steady-state STED process has a Gaussian-shaped PSF (2), which differs from our pulsed excitation, pulsed emission, time-gated detection physical implementation (see Methods Section below). The effective STED PSF is shown for ±1 radian aberrations for the 4th-8th Zernike modes, which are x astigmatism (**A**, **B**), y astigmatism (**E**, **F**), x coma (**I**, **J**), y coma (**M**, **N**), and spherical (**Q**, **R**). To the right of these PSFs are plots of the NEP-fitted diameters vs. the ground-truth diameters after fitting simulated label-filled (**C**, **G**, **K**, **O**, **S**) and surface-labeled (**D**, **H**, **L**, **P**, **T**) tubules. The color of the plotted points represents the magnitude of the respective aberration, in radians.

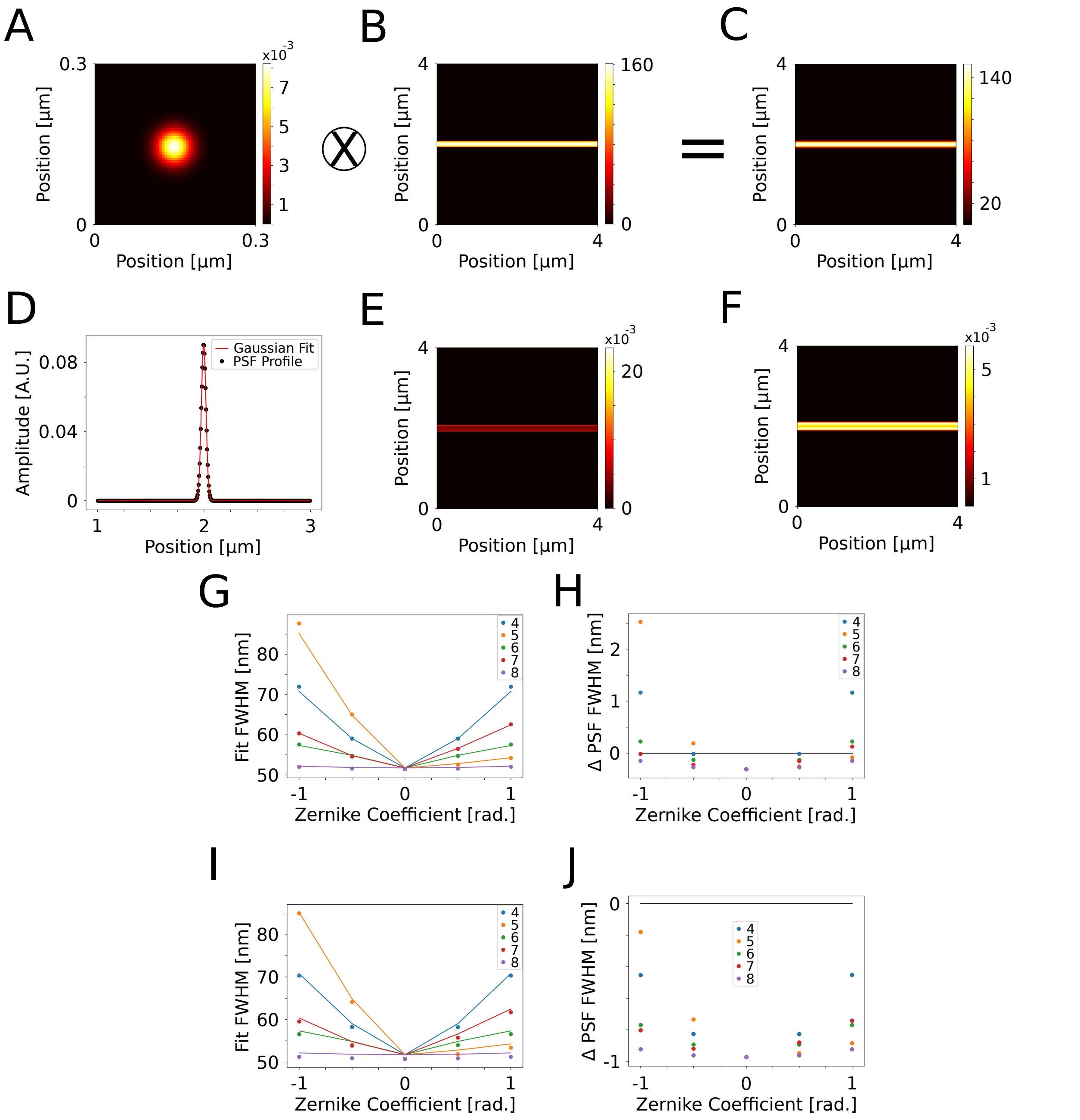

Figure S5: NEP-fitted PSF FWHM estimates are robust to ±1 radian Zernike aberrations. (**A**) Zoom in of unaberrated steady-state STED effective PSF (Gaussian profile (2), see Methods Section below), whose profile is fit in (**D**). (**B**, **E**) Fluorophore distributions for 160-nm diameter label-filled (B) and surface-labeled (E) tubules. (**C**, **F**) Tubules from (B) and (E), respectively, after being convolved with the unaberrated PSF shown in (A). (**G**, **I**) NEP-fitted PSF FWHM as a function of Zernike-mode aberration coefficient for 5 different Zernike modes (colored points) performed on label-filled (G) and surface-labeled (I) tubules ranging from 50 - 160 nm diameter. The lines represent the effective ground-truth, which is determined by fitting a Gaussian to a profile of the PSF itself, as shown in (D). (**H**, **J**) NEP-fitted PSF FWHM minus the ground-truth PSF FWHM as a function of aberration strength for both label-filled (H) and surface-labeled (J) tubules ranging from 50 - 160 nm diameter. Perfect agreement is denoted with a black line at ΔPSF FWHM = 0. Notably, agreement is within 3 nm and 1 nm for label-filled and surface-labeled tubules, respectively. As expected, the agreement is better for the surface-labeled tubules. The color of each point in (G, H, I J) represent the 4th-8th Zernike-modes describing the aberrations which correspond to x astigmatism (4), y astigmatism (5), x coma (6), y coma (7), and spherical (8).

STEDTubule_SurfaceAntibody fits

for /Users/Andrew/Downloads/NEP-fitting_supplementary/roomTemp_anti-tubulin_COS7.tif

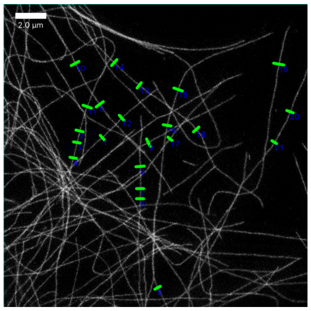

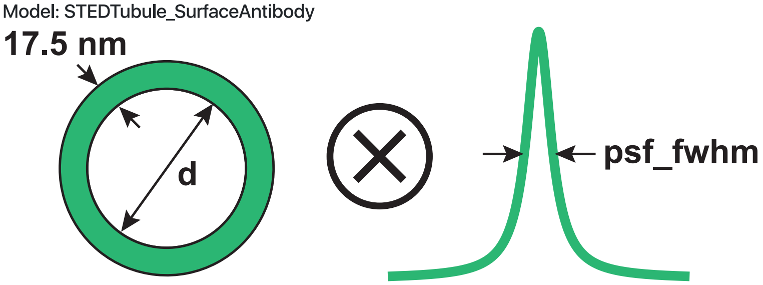

Ensemble Parameter Estimates

psf_fwhm	41.71 ± 0.42 nm

Individual fit parameters

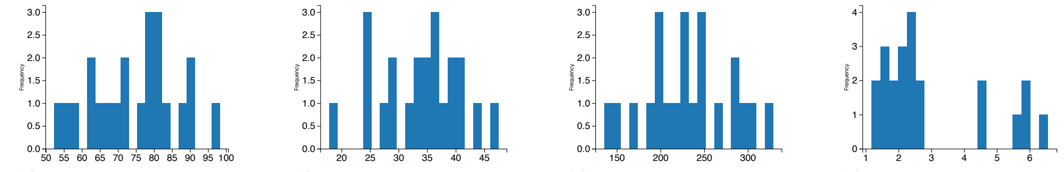

Index	amplitude	diameter	center	background
0	72.71 ± 1.96	47.44 ± 2.50	287.86 ± 1.00	1.45 ± 0.60
1	88.58 ± 2.53	17.79 ± 2.91	145.31 ± 0.75	2.44 ± 0.71
2	70.54 ± 2.61	39.21 ± 3.38	242.74 ± 1.26	2.15 ± 0.85
3	80.53 ± 1.45	28.01 ± 1.71	220.36 ± 0.54	2.44 ± 0.44

Figure S6: A (cropped) NEP-fitting report. 22 profiles were selected from the provided anti-tubulin image (see supplementary data) and then NEP-fitting was performed using the Lorentzian-convolved antibody-coated tubule model, as shown. Reports are automatically generated when calling fitting, NEP-fitting, or test ensemble values functions from the GUI. Reports are saved as html pages and can be opened with standard web browsers.

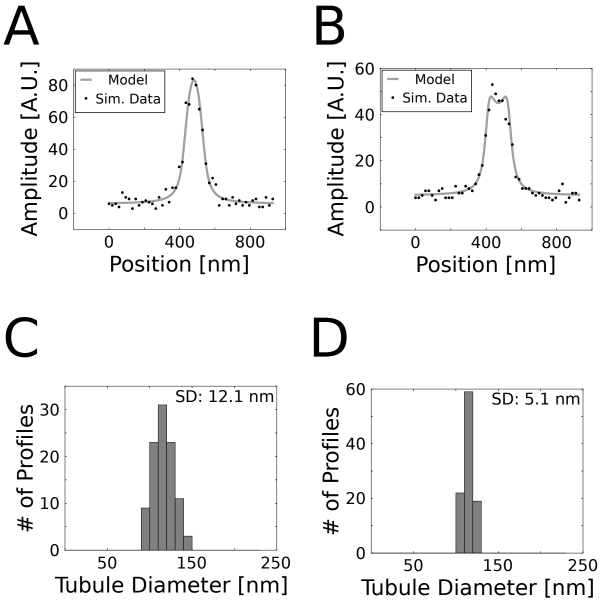

Figure S7: Variance of diameter fits for simulated 115-nm diameter tubules. (**A**, **B**) Plots of simulated profiles of a label-filled (*A*) and surface-labeled (*B*) tubule with 115-nm diameter, convolved with a 50-nm FWHM Lorentzian PSF, the expected PSF of our STED microscope. (**C**, **D**) Histograms of fitted tubule diameters for simulated 115-nm diameter label-filled (*C*) and surface-labeled (*D*) tubules. NEP fit diameters for the label-filled tubule profiles had a mean of 116 $\pm$ 12 nm (mean $\pm$ SD), and the NEP fit PSF width was 47.5 nm for these profiles. NEP fit diameters for the surface-labeled tubule profiles had a mean of 115 $\pm$ 5 nm (mean $\pm$ SD), and the NEP fit PSF width was 49.3 nm for these profiles. N=100 simulated profiles for both label-filled and surface-labeled models.

Materials and Methods

Tubule Model Functions

The model functions used in ensemble PSF fitting were derived by taking the projection of the fluorescence labeling geometry onto the xy-plane and then convolving this projection with a model of the microscope PSF. In this work we chose a Lorentzian PSF model since it resembled our experimental PSF profiles well (see Fig. S1). Derivation of the functions used was carried out in Mathematica (Wolfram Research, Champaign, IL).

Principle

Inherent to the imaging process is the convolution of the Point-Spread Function (PSF) with the structure being imaged.

$$I(x, y, z) = h(x, y, z) \otimes s(x, y, z) \tag{1}$$

where I is the resulting image, h is the PSF, and s is the fluorophore distribution. We only consider tubule cross sections because the profile of the tubule is uniform along its long-axis (y), and can therefore write the convolution as

$$I_{\mathrm{cs}}(x, z) = \int_{-\infty}^{\infty}\int_{-\infty}^{\infty} h(\tau_1, \tau_2)s(x - \tau_1, z - \tau_2)d\tau_1 d\tau_2, \tag{2}$$

where I_{cs} is the cross section of the 3D image of the tubule.

For standard STED microscopy, the full-width at half-maximum (FWHM) of the PSF along the axial dimension is significantly larger than the tubular structures we consider, such that the convolution along the axial dimension reduces to a sum. We can now write

$$P(x) = \int_{-\infty}^{\infty} h(\tau)p(x - \tau)d\tau, \tag{3}$$

where P is the line profile cross section, p is the projection of s (summed over z), and h is the 1D lateral profile of the PSF.

Annulus fluorophore distribution

The cross section of a surface-labeled tubule can be taken to be an annulus, where we assume that the fluorophores are uniformly distributed between the inner and the outer radius. Again, due to the large axial FWHM of the STED PSF, the convolution along the axial dimension reduces to a sum. We calculate this sum by considering half of an annulus, and subtracting the z position of the inner radius edge, $z_i(x)$, from the outer radius edge, $z_o(x)$,

$$z_i(x) = r \sin(\theta_i), \tag{4}$$

and

$$z_o(x) = R \sin(\theta_o), \tag{5}$$

respectively, where R is the outer radius of the annulus, r is the inner radius, $\theta_o = \arccos\left(\frac{x}{R}\right)$, and $\theta_i = \arccos\left(\frac{x}{r}\right)$. Note that outside of the inner or outer radius, respectively, $z_i(x)$ and $z_o(x)$ switch from purely real-valued to purely imaginary, which can be easily accounted for later, since the PSF they will be multiplied by in the convolution is purely real-valued. We can now write the projection of the annulus simply as

$$p_{\mathrm{annulus}}(x) = \frac{2\left(z_o(x) - z_i(x)\right)}{\pi\left(R^2 - r^2\right)}, \tag{6}$$

where the factor of two accounts for the top and bottom halves of the annulus, and we have sum-normalized $p_{\mathrm{annulus}}(x)$. The Lorentzian function in 1D is given by

$$L(x) = \frac{1}{2\pi}\frac{\gamma}{x^2 + \left(\frac{\gamma}{2}\right)^2}, \tag{7}$$

where γ is the FWHM. Substituting $L(x)$ for the 1D PSF, we can determine the line profile intensity of an annulus structure imaged with a Lorentzian PSF by evaluating

$$P_{\text{annulus}}(x) = \int_{-\infty}^{\infty} L(\tau) p_{\text{annulus}}(x - \tau) d\tau. \quad (8)$$

The integral in equation 8 can be evaluated using Cauchy's residue theorem,

$$\oint_C f(\tau) d\tau = 2\pi i \sum_j \text{Res}_j, \quad (9)$$

where C is a closed contour and Res_j are the residues of the poles within C. Our integrand, $L(\tau)p_{\text{annulus}}(x-\tau)$, has simple poles at $\tau = \pm i\gamma/2$. We therefore choose C to be the semicircular contour shown in figure S8, where we integrate along the real axis from $\tau = -\infty$ to $\tau = \infty$, and then around the arc of $\tau = \lim_{R'\to\infty} R'e^{i\theta}$ from $\theta = 0$ to $\theta = \pi$, which encloses the pole at $\tau = i\gamma/2$.

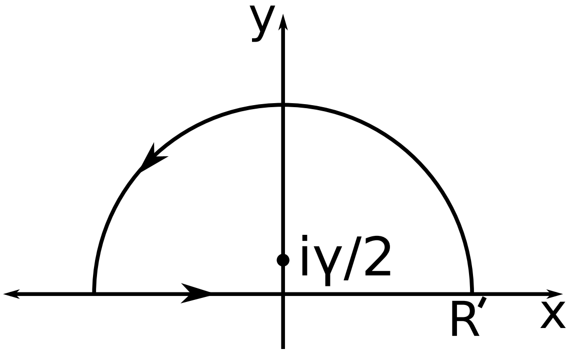

Figure S8: The contour C chosen to integrate over in the application of equation 9.

Equation 9 can now be expanded to

$$\oint_C L(\tau) p_{\text{annulus}}(x - \tau) d\tau = 2\pi i \sum_j \text{Res}_j \quad (10)$$

$$= \int_{-\infty}^{\infty} L(\tau) p_{\text{annulus}}(x - \tau) d\tau + \lim_{R'\to\infty} \int_0^{\pi} L(R'e^{i\theta}) p_{\text{annulus}}(x - R'e^{i\theta}) d\theta \quad (11)$$

$$= P_{\text{annulus}}(x) + \int_0^{\pi} L(R'e^{i\theta}) p_{\text{annulus}}(x - R'e^{i\theta}) d\theta \quad (12)$$

$$= 2\pi i \text{Res}_{\tau = i\gamma/2}. \quad (13)$$

Conveniently, $\lim_{R'\to\infty} L(R'e^{i\theta}) p_{\text{annulus}}(x - R'e^{i\theta}) = 0$, so $P_{\text{annulus}}(x) = 2\pi i \text{Res}_{\tau=i\gamma/2}$. The residue $\text{Res}_\tau = i\gamma/2$ can be calculated as $\left.\frac{g(\tau)}{h'(\tau)}\right|_{\tau=i\gamma/2}$ where g is the numerator of $L(\tau)p_{\text{annulus}}(x-\tau)$, and h' is the derivative of the denominator with respect to τ (3). This yields

$$P_{\text{annulus}}(x) = \frac{1}{\pi(r^2 - R^2)} \left[\sqrt[4]{16x^2\gamma^2 + (4r^2 - 4x^2 + \gamma^2)^2} \cos\left(\frac{1}{2} \arctan\left(4r^2 - 4x^2 + \gamma^2, 4x\gamma\right)\right) \right. \quad (14)$$

$$\left. - \sqrt[4]{16x^2\gamma^2 + (4R^2 - 4x^2 + \gamma^2)^2} \cos\left(\frac{1}{2} \arctan\left(4R^2 - 4x^2 + \gamma^2, 4x\gamma\right)\right) \right], \quad (15)$$

where $\arctan(x, y)$ is the quadrant-respecting inverse tangent function (referred to as $\arctan 2$ in several programming languages). Note that we only consider the real part of $P_{\text{annulus}}(x)$, as the imaginary component was introduced by not defining $p_{\text{annulus}}(x)$ as a piecewise function. We verified this approach by performing the convolution in Mathematica using a piecewise-defined $p_{\text{annulus}}(x)$, which achieved the same result.

Label-filled Model Function

In order to derive a label-filled model function, which can be used to model a lumen-labeled ER tubule, we have two options. The first approach would be to follow the same steps as above using the projection of a circle, which is a semi-circle

$$p_{\text{circle}}(x) = \sqrt{(R-x)(R+x)}, \tag{16}$$

where R is the radius. However, we can also simply take the limit as the inner radius of the annulus model function goes to zero, which yields

$$P_{\text{circle}}(x) = \int_{-\infty}^{\infty} L(\tau) p_{\text{circle}}(x-\tau) d\tau \tag{17}$$

$$= \lim_{r\to 0} P_{\text{annulus}}(x) \tag{18}$$

$$= \frac{1}{\pi R^2}\left[-\sqrt[4]{(4x^2+\gamma^2)^2}\cos\left(\frac{1}{2}\arctan\left(\gamma^2-4x^2, 4x\gamma\right)\right)\right. \tag{19}$$

$$\left. + \sqrt[4]{16x^2\gamma^2 + (4R^2-4x^2+\gamma^2)^2}\cos\left(\frac{1}{2}\arctan\left(4R^2-4x^2+\gamma^2, 4x\gamma\right)\right)\right]. \tag{20}$$

Gaussian-Convolved Model Functions

Confocal PSFs and PSFs for some STED modalities are better approximated by a Gaussian rather than a Lorentzian (1). While this is not the case for our experimental data, our steady-state STED simulations yield a Gaussian PSF (see Fig. S5D), as expected (2).

The (normalized) Gaussian function in 1D is given by

$$G(x) = \frac{\exp\left(-\frac{x^2}{2\sigma^2}\right)}{\sqrt{2\pi}\sigma}. \tag{21}$$

We can then write the model function for a label-filled tubule convolved with a Gaussian PSF as

$$\tilde{P}_{\text{circle}}(x) = \int_{-\infty}^{\infty} G(x-\tau) p_{\text{circle}}(\tau) d\tau, \tag{22}$$

where the tilde has been introduced to denote the Gaussian PSF model. We expanded the semicircle with a Fourier cosine series, which is given by

$$p_{\text{circle}}(x) = R\left(\frac{\pi}{2} + \sum_n \frac{(-1)^n J_1(n\pi)\cos\left(\frac{\pi n(x-R)}{R}\right)}{n}\right), \tag{23}$$

where J_1 is a Bessel function of the first kind (4) and we have not yet made any approximations.

Fortunately, convolutions are linear operators, so we can split up our convolution integral into an integral for each term in the expansion,

$$\tilde{P}_{\text{circle}}(x,R) = \int_{-R}^{R} G(x-\tau) R\left(\frac{\pi}{2} + \sum_n \frac{(-1)^n J_1(n\pi)\cos\left(\frac{\pi n(\tau-R)}{R}\right)}{n}\right) d\tau \tag{24}$$

$$= \int_{-R}^{R} G(x-\tau)\frac{\pi R}{2} d\tau + \sum_n \left[\int_{-R}^{R} G(x-\tau)\frac{R(-1)^n J_1(n\pi)\cos\left(\frac{\pi n(\tau-R)}{R}\right)}{n} d\tau\right] \tag{25}$$

$$= \Lambda_\circ + \sum_n \Lambda_n, \tag{26}$$

where

$$\Lambda_\circ = \frac{1}{4}\pi R\left(\operatorname{erf}\left(\frac{R+x}{\sqrt{2}\sigma}\right) - \operatorname{erf}\left(\frac{x-R}{\sqrt{2}\sigma}\right)\right), \tag{27}$$

and

$$\Lambda_n = \frac{R(-1)^n J_1(n\pi) e^{-\frac{\pi n\left(\pi n\sigma^2 + 2iR(R+x)\right)}{2R^2}}}{4n} \times \tag{28}$$
$$\left[\mathrm{erf}\left(\frac{\lambda + R^2 - Rx}{\sqrt{2}R\sigma}\right) + \mathrm{erf}\left(\frac{R(R+x)-\lambda}{\sqrt{2}R\sigma}\right) + e^{\frac{2i\pi nx}{R}}\left(\mathrm{erf}\left(\frac{-\lambda + R^2 - Rx}{\sqrt{2}R\sigma}\right) + \mathrm{erf}\left(\frac{R(R+x)+\lambda}{\sqrt{2}R\sigma}\right)\right)\right].$$

Where erf is the Gauss error function and we have introduced $\lambda = i\pi n\sigma^2$. Again leveraging the linear nature of convolutions, we can easily define the Gaussian-convolved annulus model function by subtracting two Gaussian-convolved circles where the circles have different radii.

$$\tilde{P}_{\mathrm{annulus}}(x) = \tilde{P}_{\mathrm{circle}}(x, R) - \tilde{P}_{\mathrm{circle}}(x, r), \tag{29}$$

where $r < R$.

We then approximate $\tilde{P}_{\mathrm{circ}}(x)$, and $\tilde{P}_{\mathrm{annulus}}(x)$ by truncating each series after $n = 5$. Our approximation matches the numerical convolution quite well, but is advantageous as it avoids discrete sampling issues.

Deriving New Model Functions

Similar model functions can be derived for other target structures and PSF model functions, with the caveat that they might not be as simple as the tubule models above. Tubules and other linear structures represent an easy class of structure to model because the cross-section is relatively uniform along the long-axis of the tubule, allowing the convolution to be ignored along that direction, and the model function to be generated only considering a single dimension. Other geometries do not allow this reduction in dimensionality, and the convolution integrals must be performed in 2 or 3D. This makes analytic model functions for STED microscopy, in particular, difficult, as 2D Lorentzians cannot be analytically normalized. In the absence of a closed analytic form, NEP fitting can be performed using numeric model functions albeit with significantly poorer computational speed.

Microtubule Simulations

Microtubule line profiles were simulated using a Lorentzian-convolved annulus model function, where the annulus had an inner diameter of 25 nm and outer diameter of 60 nm to account for a dense primary- and secondary-antibody coat (5). The center position of each microtubule was randomly varied at the sub-pixel level to avoid aliasing, and the values generated from the model were then used as expectation values in generating and sampling Poisson distributions to add shot noise to the model. The amplitude and background levels were chosen such that the signal-to-noise ratio is comparable to our fixed-cell microtubule images. The FWHM of the Lorentzian-shaped PSF was varied between 20 and 100 nm, and 50 microtubule line profiles were simulated with each PSF width.

ER Tubule Simulations

ER tubule line profiles were simulated using both label-filled and surface-labeled tubule model functions, where the model function describes the expected shape of a line profile drawn perpendicular to the long axis of a straight region of tubule. The fluorophore distribution for the surface label was taken to be an annulus of 115 nm inner diameter projected onto a line, with outer diameter 124 nm, where the 4.5 nm thickness is to account for the SNAP-tag and organic dye molecule, which were both assumed to be globular in estimating their diameters (6). The label-filled fluorophore distribution was modeled as a circle of 115 nm diameter projected onto a line. The fluorophore distributions for each model were convolved with a Lorentzian of 50 nm FWHM to emulate the microscope resolution. First, 100 profiles of each model were simulated, with their center positions randomly varied at the sub-pixel level to avoid aliasing. The intensity values generated by each model were used as expected values in generating and sampling Poisson distributions to add shot noise levels comparable to the SNAP-KDEL and SNAP-Sec61β live-cell images contained in this work. Ensemble fitting was then performed on the tubules corresponding to each model, and the fitted diameters were plotted in histograms (Fig. S1). Second, we simulated profiles at various signal-to-noise levels. The profiles were

generated in the same way, except that the pre-shot-noise background was varied from 0 to 300, while the amplitude was kept constant at 100. Due to the nature of Poisson statistics, this generates tubules with substantially different noise levels. 100 line profiles were simulated at each noise level.

Fitting

Fitting was performed in PYME using the Scipy package, specifically the Levenberg-Marquardt and Nelder-Mead minimization algorithms. For standard, non-NEP fitting, profile fits were performed with the Scipy package Levenberg-Marquardt implementation. All initial parameter guesses were automatically estimated. The background (offset from zero) was estimated to be the minimum intensity value of the profile, the amplitude of the profile was estimated to be the maximum intensity value minus the background, and the center position was estimated to be the position of the maximum intensity value. The FWHM of the Gaussian and Lorentzian functions, and the tubule diameter and PSF FWHM of the Lorentzian-convolved model functions were all estimated to be the FWHM of the profile, which was determined by counting the number of pixels with intensity values above the background plus half of the amplitude.

For NEP fitting, which was only applied using the Lorentzian-convolved model functions, the inner loop fitting was performed similarly to the standard fitting, with the only difference being that the inner loop did not try to optimize the PSF FWHM, and instead took this value as an input parameter parsed by the outer loop. The inner loop returned the mean of the mean squared errors (mean MSE) taken over each of the individual tubules fits, and this value was minimized in the outer loop fit using a Nelder-Mead minimization, where the only parameter directly controlled by the Nelder-Mead minimization was the PSF FWHM. In order to estimate the uncertainty of the PSF FWHM fit, the result from the Nelder-Mead minimization was passed as an initial guess to a Levenberg-Marquardt minimization to approximate the variance of the estimate. We used the Nelder-Mead algorithm for the primary parameter estimation because we found it to converge faster. In order to facilitate the estimation of the variance, the inner loop passed the Levenberg-Marquardt minimization an array of the residuals appended from each of the tubule profile fits. The variance estimate is calculated by multiplying the residual variance by the jacobian about the fit result.The standard deviation, however, is often below 1 nm for NEP fitting, which we take to be an underestimate of the uncertainty in the measurement.

Initial Guess Simulations

The simulations testing whether the fitting is robust to poor initial guesses used label-filled tubules, as the PSF width and tubule diameter are more highly coupled than for the surface-labeled tubules (see Fig. 3E, F, Fig. S7C, D, and Fig. S2D, E, I, J), suggesting that label-filled tubules would be more susceptible to poor initial guesses. The initial guesses for the fitting parameters were calculated as described above, however they were modified by a multiplicative factor we refer to as the 'guess multiplier'. The exception was the center position fit parameter, which was not affected by the guess multiplier, but instead was shifted from its initial calculation by one PSF FWHM (50 nm) for all simulated profiles. N=100 tubules of 115 nm diameter were simulated for each guess multiplier, using a PSF FWHM of 50 nm. Poisson noise was added to each profile, an example of which is shown in figure S3A.

Aberration Simulations

To simulate the effects of aberrations on NEP-fitting measurements, we simulated aberrated STED PSFs as follows. We used a vectorial, Fourier optics based, propagation strategy to generate 3D excitation, depletion, and detection PSFs from an arbitrary pupil function and took the in-focus slice as a 2D PSF which was convolved with a 2D projection of the target structures. PSFs were simulated using a 1.4 NA objective, with 650 nm excitation, 775 nm depletion, detection after a 1 Airy unit pinhole, and a steady-state exponential saturation model with a saturation factor of $I/I_{\text{sat}} = 25$. We assumed the depletion donut was slightly imperfect, and that the "zero" at the center was therefore 1% of the depletion profile maximum. This normalization was then

$$d_{\text{norm}}(x, y) = (1 - \delta)\,\frac{d(x, y) - d(0, 0)}{\max d(x, y) - d(0, 0)} + \delta, \qquad (30)$$

where $(x,y) = (0,0)$ is the center of the depletion profile $d(x,y)$ and we chose a δ of 0.01. The effective STED PSF was then calculated as

$$h(x,y) = h_{\text{excitation}}(x,y)h_{\text{detection}}(x,y)e^{-d_{\text{norm}}(x,y)I/I_{\text{sat}}}. \tag{31}$$

Aberrations were applied to the excitation and depletion PSFs, as well as the detection PSF (before being convolved with the pinhole). The aberrations were applied independently so each effective PSF corresponds to a single Zernike mode aberration of a given amplitude ranging from -1 rad to 1 rad. These PSFs were simulated over 4 µm x 4 µm areas with 5 nm pixel sizes. They fit nicely to a Gaussian profile, as expected for the steady-state continuous-wave assumption implicit in equation 31.

The fluorophore distribution of the label-filled and surface-labeled tubules were generated for 12 tubules evenly spaced in diameters from 50 nm to 160 nm. The thickness of the annulus for the surface-labeled tubules was set to be 4.5 nm to represent SNAP-tag labeling, and both surface-labeled and label-filled tubules were simulated with 5 nm pixel sizes over a 4 µm x 4 µm. For each aberrated PSF tested, these 12 tubules were convolved with the PSF, then a profile orthogonal to the long axis was extracted. These 12 profiles then constituted the ensemble of profiles for NEP-fitting for that PSF.

The aberrated PSFs were additionally fit separate of any tubule convolution in order to determine the ground-truth to compare the NEP-fitting results against. The 2D PSF was summed along the same dimension as the long-axis of the tubule to generate the 1D profile which was then fit to a 1D Gaussian. The sum was performed rather than a slicing operation in order to account for the two-dimensional convolution.

Cell Culture

COS-7 (ATCC, CRL-1651) cells were grown in a standard mammalian cell incubator with 5% CO_2 environment using phenol red free DMEM (Thermo Fisher Gibco) or DMEM/F-12 (Thermo Fisher Gibco) media supplemented with 10% FBS (Thermo Fisher Gibco). Cells were transfected by electroporation using a Super Electroporator NEPA21 Type II (Nepa Gene). Electroporation cuvettes with a 2 mm gap were loaded with 10^6 cells suspended in Opti-MEM (Thermo Fisher Gibco) and 2.5-10 µg DNA, depending on the desired expression level. Transfected cells were imaged 12-48 hours after electroporation.

Microtubule Samples

Microtubule samples were prepared using the method described by Huang et al (2016) (7). Briefly, COS-7 cells were grown on coverglass and pre-extracted using saponin before fixation with 3% paraformaldehyde + 0.1% glutaraldehyde. Mouse anti-α-tubulin antibody (Sigma-Aldrich, T5168) was used to label microtubules. A goat anti-Mouse antibody labeled with Atto647N (Sigma-Aldrich) was used as a secondary antibody. Samples were mounted in Prolong Diamond Antifade Mountant (Thermo Fisher Scientific) and imaged at room temperature.

ER Samples

The SNAP-Sec61β images used are a subset of images from a broader study on ER morphology (8). COS-7 cells were electroporated with either SNAP-Sec61β (9) or SNAP-KDEL (8) and plated in glass-bottom dishes (MatTek, 35 mm, no. 1.5). SNAP-tagged proteins were labeled immediately before imaging with 1 µM SNAP-Cell 647-SiR (New England Biolabs, S9102S) according to manufacturer's instructions. Living cells were imaged with 5% CO2, in Live Cell Imaging Solution (Thermo Fisher Scientific) supplemented with 15 mM D-glucose, and at 37 C using a stage incubator and objective heater.

STED Microscopy

Images were acquired using a Leica SP8 STED 3X equipped with a Onefive Katana-08HP pulsed laser as a depletion light source (775 nm wavelength) and a SuperK Extreme EXW-12 (NLT Photonics) pulsed white light laser as an excitation source. All images were acquired using a HC PL APO 100x 1.40 NA Oil CS2 objective. For living cells, imaging was performed with 8000 Hz line-scan speed and the cells were kept at 37 C with 5% CO_2. Fixed cells were imaged with a 1000 Hz line-scan speed, at room temperature. Images

were acquired using 16 line averages. All samples were imaged with 633 nm excitation and 775 nm depletion wavelengths, with depletion laser power of 110.6 mW for all images of ER. Emission light between 650-750 nm was collected using a detection gate set to 0.3-6 ns, on a HyD hybrid detector. The pinhole was set to 1 Airy unit.

Depletion Power Measurements

STED laser powers on the Leica SP8 STED 3X were measured using a microscope slide power meter sensor head (ThorLabs, S170C) with a digital handheld optical power meter console (ThorLabs, PM100D). Laser power at 775 nm depletion wavelength was measured using microscope settings to slowly scan a very small region with minimal beam blanking, which allowed us to detect the depletion laser light as a point rather than a scanned line. A 8192 x 8192 pixel region was scanned with 10 Hz line-scan speed, at minimum pixel size, and using bidirectional scanning at room temperature. These settings effectively scanned a 2.42 x 2.42 µm region with 295.68 pm sized pixels and 3.05 µs pixel dwell time. Laser power detected with these setting is equivalent to using the 'bleachpoint tool', as measured for an pulsed excitation laser (SuperK Extreme EXW-12, NKT Photonics) set at 660 nm wavelength. The depletion laser powers used in this work, 27.7 mW, 55.6 mW, and 110.6 mW, correspond to AOTF settings in the Leica software of 10%, 20%, and 40%, respectively.

Box Plots

The whiskers of all box plots represent the 10th and 90th percentiles of each distribution, the colored boxes cover the interquartile range, and the center line in each box denotes the median. Box plots and swarm plots were generated using the Seaborn Python package.

Software User Guide

Installation

NEP-fitting has flexible installation routes, but it is recommended to begin by installing the anaconda Python 2.7 distribution. Afterwards, the NEP-fitting plug-in and PYME can be installed by either using Anaconda Navigator (completely GUI-based) or by entering `conda config --add channels david_baddeley` followed by `conda install -c barentine nep-fitting` to the terminal. To install using Anaconda Navigator, open Navigator, add the channel by clicking the 'Add' button and entering 'barentine'. Do the same for the channel 'david_baddeley'. Now, click the 'environments' tab, search nep-fitting, select it, and click 'add'. Installation for these routes can be verified by running `STEDFitter` on the terminal/command prompt.

For contributing to or modifying the NEP-fitting plug-in, it is recommended to install using setuptools. Instructions for this are available at `github.com/bewersdorflab/nep-fitting`. If you install via setuptools, you will need to manually load the NEP-fitting plug-in after opening dh5view rather than simply calling `STEDFitter`; in this case, run `dh5view`, select an image to load, and select 'sted_psf_fitting' from the 'modules' drop-down menu.

Extracting Profiles

Extracting profiles begins by opening an image by `STEDFitter` in the terminal/command prompt. Click the square button with a diagonal line through it (1) to begin selecting line profiles. Click the starting point of the line, hold down, and release when the mouse is over the desired end-point. Press 'Add' (2). You should now see the line in the profile list at left. You can toggle the visibility of any line profile(s) by selecting it in the profile list, and clicking 'Show/Hide' (3). Similarly, one can remove a profile by selecting it in the list and clicking 'Delete' (4). The number of pixels to average the profile over (perpendicular to the line) can be set by typing a value into the 'Line width' box (5) and pressing enter (or clicking else-where). This updates the width for all profiles.

Once you have extracted the desired line profiles, you can save your profiles by clicking 'Save' (6). If you would like to analyze profiles from multiple images simultaneously, as is done in this work, you may append

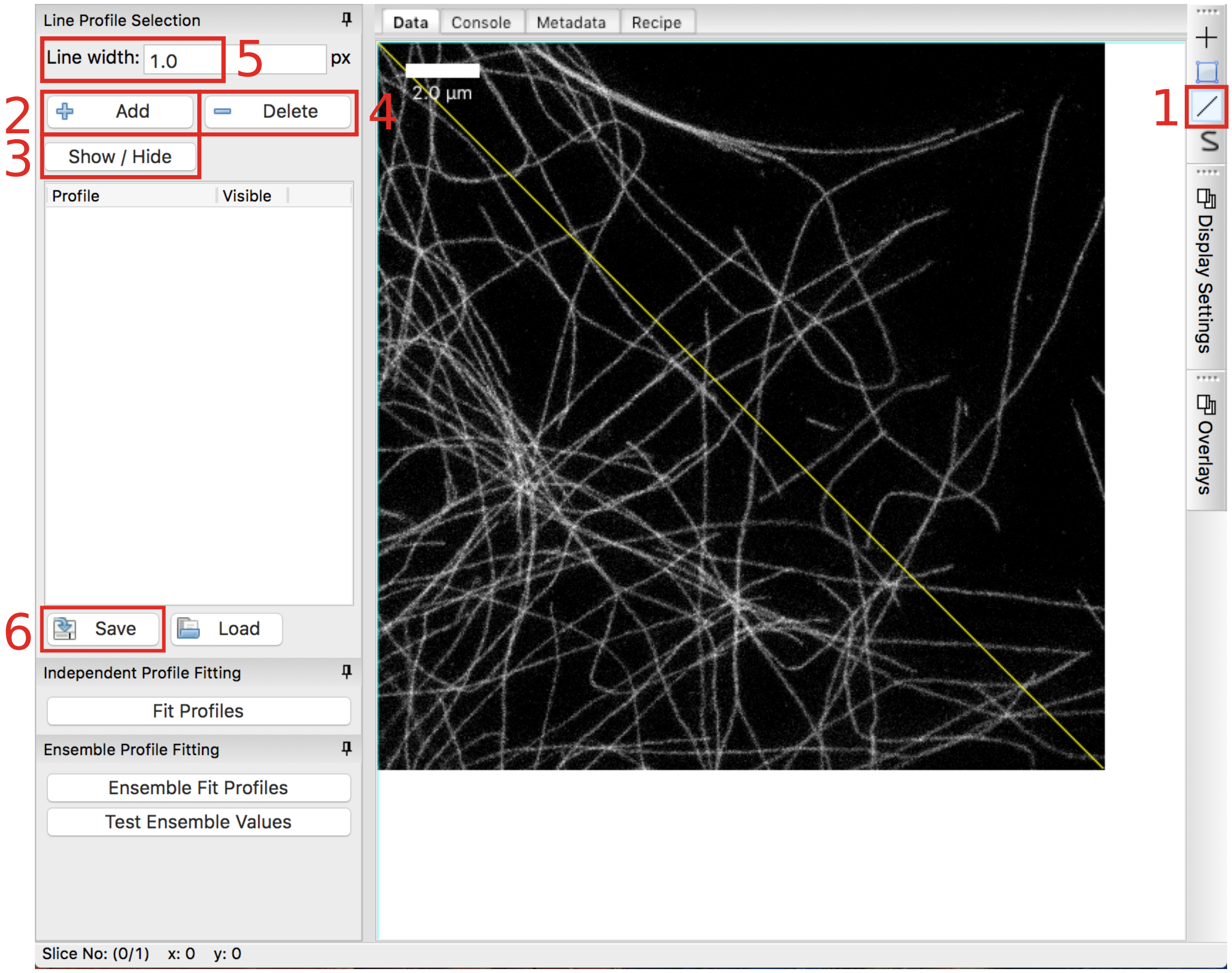

line profiles to the same file by clicking 'Save' and selecting the same file to save to. In this case, you will have to acknowledge a prompt which checks if you in fact would like to append to the previously written file.

Fitting Profiles

Line profiles can be extracted, as described above, or loaded using the 'Load' button (7) and selecting the HDF or JSON file containing the profiles in the resulting dialogue. After this is performed, standard, non-ensemble fits can be carried out by clicking 'Fit Profiles'(8), and NEP-fitting can be performed by clicking 'Ensemble Fit Profiles' (9). Each of these will open dialog boxes, asking which model to use during fitting, and potentially an initial guess for the PSF width. Upon completion of these fits, you will be prompted to save the fit results, after which a report will automatically be generated in HTML and opened as a new tab in your web browser.

The procedure for manually testing various PSF widths is quite similar to performing the fits, and is instead accomplished by clicking 'Test Ensemble Values' (10). You will be asked to enter the test values in a dialogue box, after which the values will be tested, and you will be prompted to save the report. After saving the report, it will again open as a new tab in your web browser.

Fitting, NEP-fitting, and ensemble testing can additionally be performed using the PYME bakeshop. The bakeshop can be opened by running `bakeshop`, and a recipe can be constructed either by adding recipe tiles manually, adding them using the 'Add Module' button, loading a saved recipe, or copying the text similar to the following (adjusted for the desired fit model, etc) into the box at right:

```
- nep_fits.EnsembleFitProfiles:
```

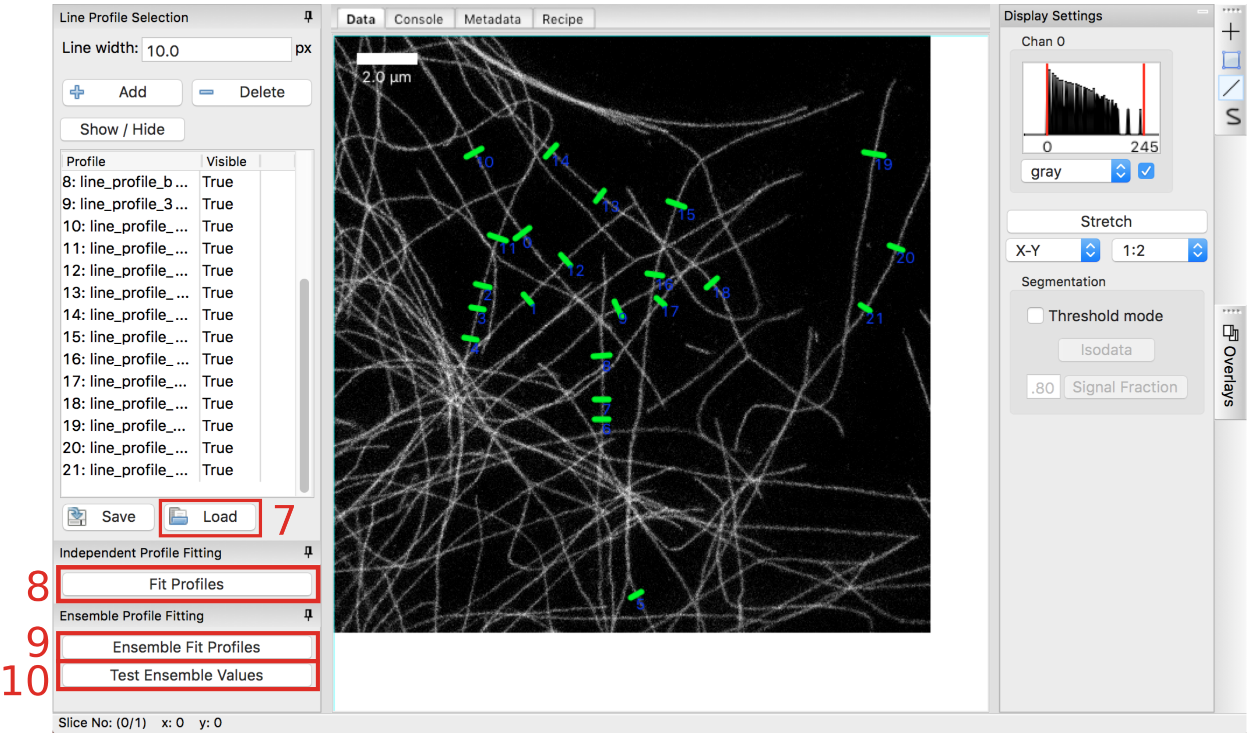


```
    ensemble_parameter_guess: 50.0
    fit_type: STEDTubule_Lumen
    hold_ensemble_parameter_constant: false
    inputName: line_profiles
    outputName: fit_results
- output.HDFOutput:
    filePattern: '{output_dir}/{file_stub}.hdf'
    inputVariables:
      fit_results: fitResults
    scheme: File
```

The input file(s) can be selected using filename patterns and clicking ‘Get Matches’. Finally, select the output folder by manual entry or through the ‘Browse’ dialogue, and click ‘Bake’.

References

[1] Vicidomini, G., A. Schönle, H. Ta, K. Y. Han, G. Moneron, C. Eggeling, and S. W. Hell, 2013. STED Nanoscopy with Time-Gated Detection: Theoretical and Experimental Aspects. *PLoS One* 8.

[2] Harke, B., J. Keller, C. K. Ullal, V. Westphal, A. Schönle, and S. W. Hell, 2008. Resolution scaling in STED microscopy. *Optics Express* 16:4154.

[3] Riley, K., M. Hobson, and S. Bence, 2006. Mathematical methods for physics and engineering. Cambridge University Press, third edition.

[4] Weisstein, E. W. Fourier Series–Semicircle. From MathWorld—A Wolfram Web Resource. `http://mathworld.wolfram.com/FourierSeriesSemicircle.html`.

[5] Weber, K., P. C. Rathke, and M. Osborn, 1978. Cytoplasmic microtubular images in glutaraldehyde-fixed tissue culture cells by electron microscopy and by immunofluorescence microscopy. *Proceedings of the National Academy of Sciences of the United States of America* 75:1820–4.

[6] Erickson, H. P., 2009. Size and shape of protein molecules at the nanometer level determined by sedimentation, gel filtration, and electron microscopy. *Biological Procedures Online* 11:32–51.

[7] Huang, F., G. Sirinakis, E. S. Allgeyer, L. K. Schroeder, W. C. Duim, E. B. Kromann, T. Phan, F. E. Rivera-Molina, J. R. Myers, I. Irnov, M. Lessard, Y. Zhang, M. A. Handel, C. Jacobs-Wagner, C. P. Lusk, J. E. Rothman, D. Toomre, M. J. Booth, and J. Bewersdorf, 2016. Ultra-High Resolution 3D Imaging of Whole Cells. *Cell* 166:1028–1040.

[8] Schroeder, L. K., A. E. S. Barentine, S. Schweighofer, D. Baddeley, J. Bewersdorf, and S. Bahmanyar, 2017. Nano-scale size holes in ER sheets provide an alternative to tubules for highly-curved membranes. *bioRxiv* .

[9] Bottanelli, F., E. B. Kromann, E. S. Allgeyer, R. S. Erdmann, S. Wood Baguley, G. Sirinakis, A. Schepartz, D. Baddeley, D. K. Toomre, J. E. Rothman, and J. Bewersdorf, 2016. Two-colour live-cell nanoscale imaging of intracellular targets. *Nature Communications* 7:10778.
