# Supplementary figures and images for: Simultaneously Measuring Image Features and Resolution in Live-Cell STED Images
Source: Biophys J. 2018 Aug 4;115(6):951–6. doi: 10.1016/j.bpj.2018.07.028 (PMC6139878; doi:10.1016/j.bpj.2018.07.028)

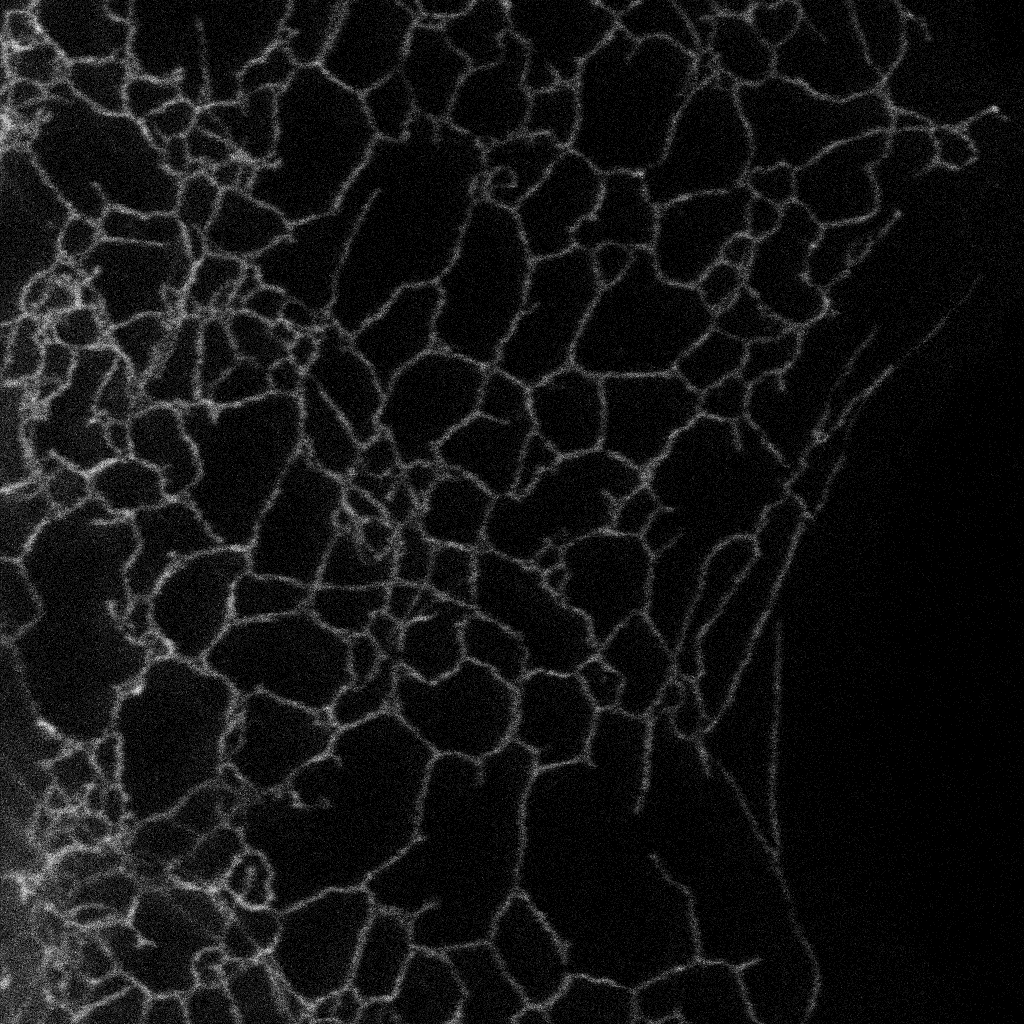

Supplement: Data S1. Supporting Data [file mmc2.zip › supplemental_data/37C_live_Snap-KDEL_COS7.tif]

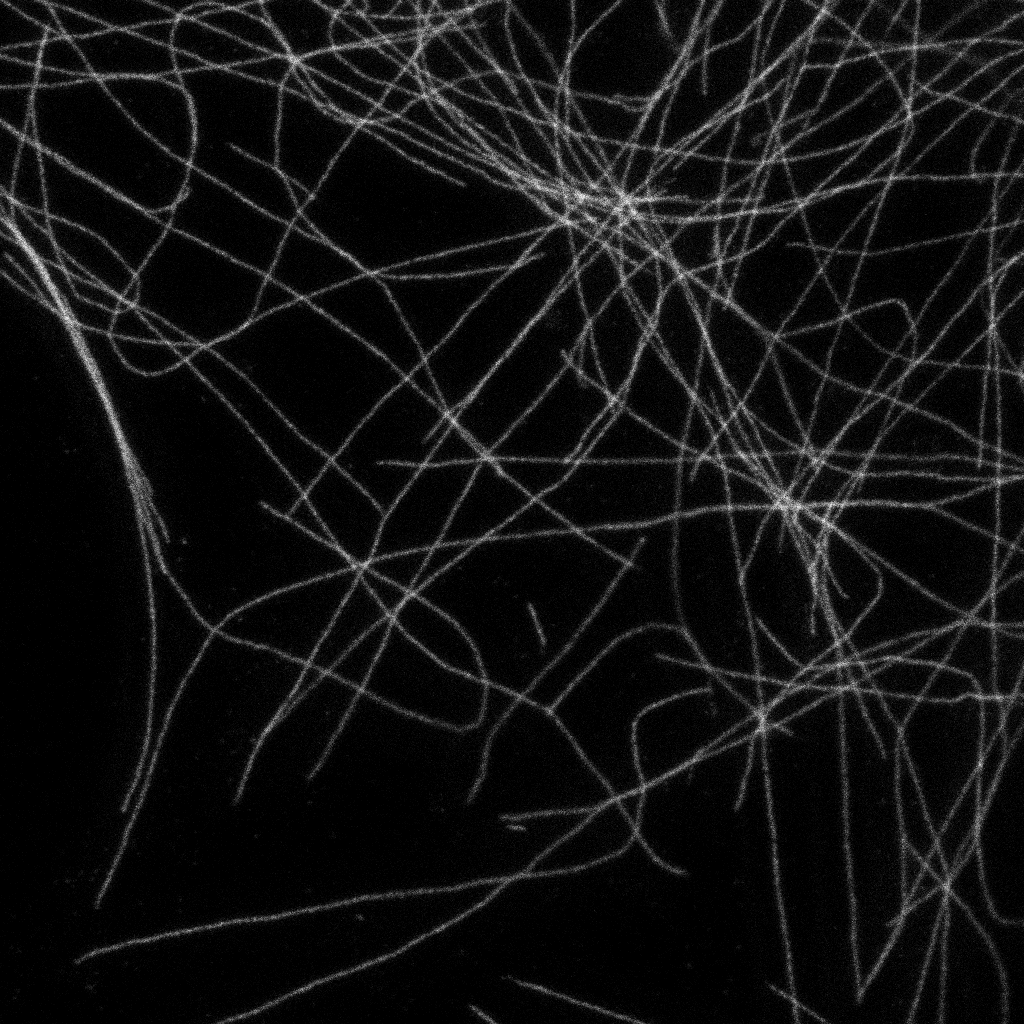

Supplement: Data S1. Supporting Data [file mmc2.zip › supplemental_data/roomTemp_anti-tubulin_COS7.tif]
