# Supplementary material for: Simultaneously Measuring Image Features and Resolution in Live-Cell STED Images
Source: Biophys J. 2018 Aug 4;115(6):951–6. doi: 10.1016/j.bpj.2018.07.028 (PMC6139878; doi:10.1016/j.bpj.2018.07.028)
Supplement: Document S2. Article plus Supporting Material [file mmc3.pdf]

Biophysical Journal
Computational Tool

Simultaneously Measuring Image Features and Resolution in Live-Cell STED Images

Andrew E. S. Barentine,[1,2] Lena K. Schroeder,[1] Michael Graff,[1] David Baddeley,[1,3,*] and Joerg Bewersdorf[1,2,*]
[1]Department of Cell Biology, Yale University School of Medicine, New Haven, Connecticut; [2]Department of Biomedical Engineering, Yale University, New Haven, Connecticut; and [3]Auckland Bioengineering Institute, University of Auckland, Auckland, New Zealand

ABSTRACT Reliable interpretation and quantification of cellular features in fluorescence microscopy requires an accurate estimate of microscope resolution. This is typically obtained by measuring the image of a nonbiological proxy for a point-like object, such as a fluorescent bead. Although appropriate for confocal microscopy, bead-based measurements are problematic for stimulated emission depletion microscopy and similar techniques where the resolution depends critically on the choice of fluorophore and acquisition parameters. In this article, we demonstrate that for a known geometry (e.g., tubules), the resolution can be measured in situ by fitting a model that accounts for both the point spread function (PSF) and the fluorophore distribution. To address the problem of coupling between tubule diameter and PSF width, we developed a technique called nested-loop ensemble PSF fitting. This approach enables extraction of the size of cellular features and the PSF width in fixed-cell and live-cell images without relying on beads or precalibration. Nested-loop ensemble PSF fitting accurately recapitulates microtubule diameter from stimulated emission depletion images and can measure the diameter of endoplasmic reticulum tubules in live COS-7 cells. Our algorithm has been implemented as a plugin for the PYthon Microscopy Environment, a freely available and open-source software.

INTRODUCTION

Fluorescence microscopy images never represent the underlying object perfectly, failing to discern details smaller than a certain size. This imperfection is described by the system's point-spread function (PSF). Knowledge of the PSF is essential when interpreting the images produced and in ensuring that quantitative measurements are accurate. For many purposes, it is sufficient to summarize the effects of the PSF in a simple resolution metric (e.g., the full-width at half maximum (FWHM)). A popular method for obtaining the PSF FWHM is extracting an intensity line profile from a fluorescent bead image and either directly measuring the FWHM or estimating it more accurately by fitting a Gaussian or Lorentzian (in the case of stimulated emission depletion (STED)) model to the profile. For diffraction-limited microscopes, beads can be regarded as point-sources because they are significantly smaller than the FWHM of the PSF (beads are typically 20–100 nm compared to the ~250 nm PSF FWHM), and the fit FWHM is taken to be that of the PSF.

When considering STED microscopy, where the PSF FWHM is typically 25–70 nm, the assumption that the PSF is much larger than the bead size is no longer valid, as beads whose size is similar to that of the PSF are often needed to achieve reasonable signal levels. The resolution in STED microscopy is also strongly affected by laser powers, sample-induced aberrations, and the depletion cross-section of the dye, which is additionally dependent on the local environment, making beads a poor proxy for the true resolution achieved when imaging cellular samples.

Measuring STED resolution on a target in the same cellular environment labeled with the same dye(s) and imaged with the same laser powers avoids these issues. Microtubules labeled with the same fluorescent dye as the final target structure are an attractive candidate that can be readily prepared. The simplest and most common labeling protocol that usually results in bright stainings is indirect immunofluorescence. Labeling the 25-nm outer diameter of a microtubule with primary and secondary antibodies results in a structure that is 60 nm in diameter as observed using electron microscopy (1). However, this is within the size range of a STED PSF, so as with beads the thickness of the structure is nonnegligible when quantifying the resolution of a microtubule image. To determine the impact finite object size has on resolution quantification using the popular

Submitted November 20, 2017, and accepted for publication July 24, 2018.
*Correspondence: d.baddeley@auckland.ac.nz or joerg.bewersdorf@yale.edu
Editor: Valentin Nagerl.
https://doi.org/10.1016/j.bpj.2018.07.028
© 2018 Biophysical Society.
This is an open access article under the CC BY license (http://creativecommons.org/licenses/by/4.0/).

Gaussian- and Lorentzian-fitting techniques, we simulated intensity line profiles perpendicular to the long axis of antibody-labeled microtubules imaged at various resolutions and fit them with Gaussian and Lorentzian functions.

We modeled the fluorophore distribution for the primary and secondary antibody labeled microtubule as an annulus of 25-nm inner diameter and 60-nm outer diameter, as measured for densely-labeled microtubules (1) (Fig. 1 *A*, *top*). For most STED microscopes, the axial (z) PSF FWHM is considerably larger (500–700 nm) than the FWHM along the lateral (xy) directions. This means that the entire cross-section of a microtubule is effectively summed along the axial dimension during imaging, producing the red dash-dot curve in Fig. 1 *A*. The imaging process is simulated by convolving the fluorophore distribution with the PSF model (Fig. 1 *A*, *teal dashed curve*). For these simulations, we modeled the PSF as a Lorentzian, a common STED PSF approximation (2,3) that represents our experimental PSF better than a Gaussian (see Fig. S1 in the Supporting Material). The resulting cross section of a microtubule imaged with a 50-nm FWHM PSF is shown in Fig. 1 *A* (*green solid curve*).

We simulated line profiles across microtubules imaged with STED resolutions ranging from 20 to 100 nm (PSF FWHM) with added shot noise and fit them with simple Lorentzian and Gaussian functions. The FWHM of the Lorentzian fits, and in particular the Gaussian fits, were substantially larger than the PSF FWHM they were simulated with (Fig. 1 *B*), confirming that simple fitting of these models does not result in an accurate resolution measure. The accuracy deteriorates, as expected, at higher resolutions (smaller PSF FWHM), making it particularly problematic for STED microscopy, in which systematic errors of 100% can easily occur.

Similar Gaussian or Lorentzian fits in which the width is interpreted as the size of an imaged structure rather than resolution are popular in various types of fluorescence (super-resolution) microscopy. Interpreting Fig. 1 *B* this way shows that this approach only yields reasonable results when the PSF is much smaller than the imaged structure. We conclude that using the FWHM of Gaussian or Lorentzian fits is an unreliable measure for both resolution and feature size quantification.

As an alternative to fitting of line profiles, Fourier ring correlation, a powerful model-free method of measuring relative resolution in images of similar objects (4,5), has been used to quantify STED resolution (6). Unfortunately, Fourier ring correlation results are highly dependent on the structure being imaged, subject to the same limitations relating to object dimensions as simple profile fits, and are not directly translatable into FWHM values.

In order to accurately determine microscope resolution or feature size from line profile cross-sections, both the microscope PSF and the geometric distribution of fluorophores on

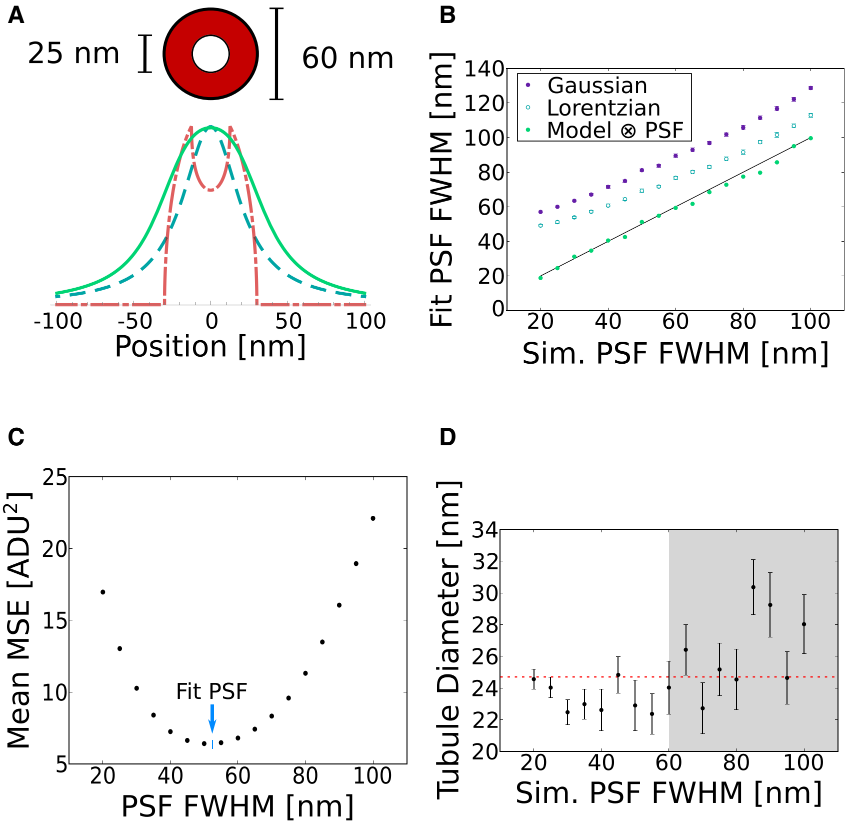

FIGURE 1 (*A*) Annulus used to model fluorophore location, where antibodies and fluorophores are bound to the surface of a 25-nm diameter microtubule (1). The red dash-dot curve represents a projection of the fluorophore distribution (summing over the axial dimension), the teal dashed curve a Lorentzian function that models the PSF, and the green solid curve the convolution of the other two. (*B*) Microtubule line-profiles were simulated at various resolutions using Lorentzian PSFs, with shot noise added before being fit with simple Gaussian (*purple*) and Lorentzian (*teal hollow*) functions. The same profiles were also fit using NEP fitting (*green*), which results in good agreement with the ground truth of the simulations (*black line*). N = 50 profiles were fit for each simulated PSF width. (*C*) Plot of mean MSE for fits performed with the Lorentzian-convolved model function at specified PSF widths on simulated microtubule profiles. These profiles were generated with a 50-nm PSF and added shot noise. NEP fitting minimizes the mean MSE with a PSF FWHM of 51.2 nm, as indicated by the blue arrow. (*D*) Plot of microtubule diameters determined by NEP fitting, where images were simulated at various resolutions (N = 50 profiles at each PSF width, error bars denote standard error of the mean). The ground truth diameter was 25 nm for all profiles, as shown by the dashed red line. The gray region of the plot indicates where the simulated PSF FWHM is larger than the antibody-coated tubule structure, which results in less accurate tubule diameter fits. To see this figure in color, go online.

the labeled structure must be modeled in the function used for fitting. Previous efforts have held-fixed one of these parameters, assuming that either the PSF or the structure size is already known and enforcing this assumption either during fitting (7) or in a simulation to quantify the FWHM bias of the fit (8). However, these approaches are limited because in biological STED microscopy, both structure size and resolution are typically unknown. Fitting both of these parameters simultaneously, on the other hand, is difficult; increases in either parameter give rise to an increased profile width, albeit with subtly different effects on profile shape. At the signal-to-noise (SNR) level typical of a single profile, it is difficult to separate the effects of the two parameters. This coupling can result in inaccurate estimates for both values. Here, we present a tool that overcomes this challenge and allows simultaneous fitting of structure size and PSF width.

Ensemble PSF Fitting

We enable robust simultaneous determination of PSF width and structure size by fitting multiple line profiles as an ensemble, exploiting prior knowledge that the PSF width should be the same for each profile. We accomplish this by performing a two-layer nested fit, such that in the inner fit, all tubules are fit with the same PSF FWHM, γ, and the mean squared error (MSE) for each tubule is reported. The MSE averaged over all tubule fits as a function of γ has a propensity to be well behaved and smooth (Fig. 1 *C*). The outer fit is then responsible for finding the value of γ which minimizes the mean MSE. This technique, which we refer to as nested-loop ensemble PSF (NEP) fitting, constrains the fit enough that accurate PSF widths and microtubule diameters can be determined, as shown in Fig. 1, *B* and *D*.

NEP fitting using the antibody-coated tubule model yielded significantly better results than fitting with plain Gaussian or Lorentzian functions, and the PSF widths calculated by the fit are in close agreement with the ground truth (Fig. 1 *B*). Simultaneously, it yielded accurate measures of the simulated microtubule diameter, 25 nm, for all simulated PSFs with FWHM equal to or less than 60 nm, which is the value at which the PSF FWHM becomes larger than the outer diameter of the antibody coat (Fig. 1 *D*). For structures whose size does not vary in a cell (e.g., microtubule diameters), the structure size can additionally be constrained as an ensemble parameter during the fit, although we did not find this to be a necessary step.

We carried out additional simulations to determine the robustness of NEP fitting on low SNR data as well as data obtained with aberrated PSFs. Not surprisingly, the variability in fit tubule diameters for individual profiles is increased at lower SNRs (Fig. S2), yet NEP fitting estimates for tubule diameter and PSF width do not suffer from systematic errors. Systematic changes in initial fit parameter estimates led to negligible differences in the fit outcomes (see Fig. S3), demonstrating the robustness of the nested fit approach. We then simulated STED PSFs with various aberrations and found that sizes of the convolved structures were still accurately determined (Fig. S4). Additionally, the PSF width estimates from NEP fitting were in good agreement with the FWHM of the simulated (aberrated) PSFs (Fig. S5).

Software and Validation

We implemented NEP fitting for STED images of label-filled or surface-labeled cylindrical structures in the PYthon Microscopy Environment (PYME). Line profiles of a user-defined width are extracted from images loaded into PYME, after which they can be fit using a variety of model functions. Alternatively, they can be saved or appended to two file formats (Hierarchical Data Format, JavaScript Object Notation) for later analysis or ensemble fitting with profiles from multiple images. Fitting performed through the graphical user interface (GUI) saves results to an Hierarchical Data Format table and generates an HTML report (see Fig. S6 for an example report). The line profile extraction GUI is shown in Fig. 2 *A*.

STED PSF width is dependent on the STED laser power with a scaling of $\alpha/\sqrt{1+I/I_s}$, where I is the depletion intensity and α and I_s are constants (9). To test the efficacy of ensemble fitting on real data, we imaged primary and secondary antibody-labeled microtubules using a Leica SP8 STED 3X microscope (Leica Microsystems, Wetzlar, Germany) at different STED laser powers. Fig. 2 *C* shows that our fit is responsive to changes in PSF size and can reproduce the expected scaling of PSF width with respect to the STED laser power.

To determine if our NEP fitting approach in which the PSF width is constrained to a single, global value for all profiles is beneficial, we compared its results with those obtained when both PSF width and tubule diameter were optimized on a per-profile basis. We performed this comparison on images recorded with 111-mW depletion power. Although fits performed without an ensemble PSF have more degrees of freedom and therefore usually yield smaller residuals, they come at the expense of accuracy in the measured values. This can be seen in Fig. 2 *D*, where the standard least squares fit results in an average microtubule diameter of 30 $\pm$ 13 nm (mean $\pm$ standard deviation (SD)), compared to the NEP-fitted value of 28 $\pm$ 8 nm (mean $\pm$ SD). NEP fitting, with its global PSF constraint, improves the measurement of the microtubule diameter, as evident from the reduced spread in tubule diameters and average value closer to the expected 25 nm. The PSF FWHM of standard least squares fitting was 45 $\pm$ 10 nm (mean $\pm$ SD), compared to the NEP-fitted value of 44 nm.

We note that fitting a single line profile would not provide a reliable measure of either PSF FWHM or tubule

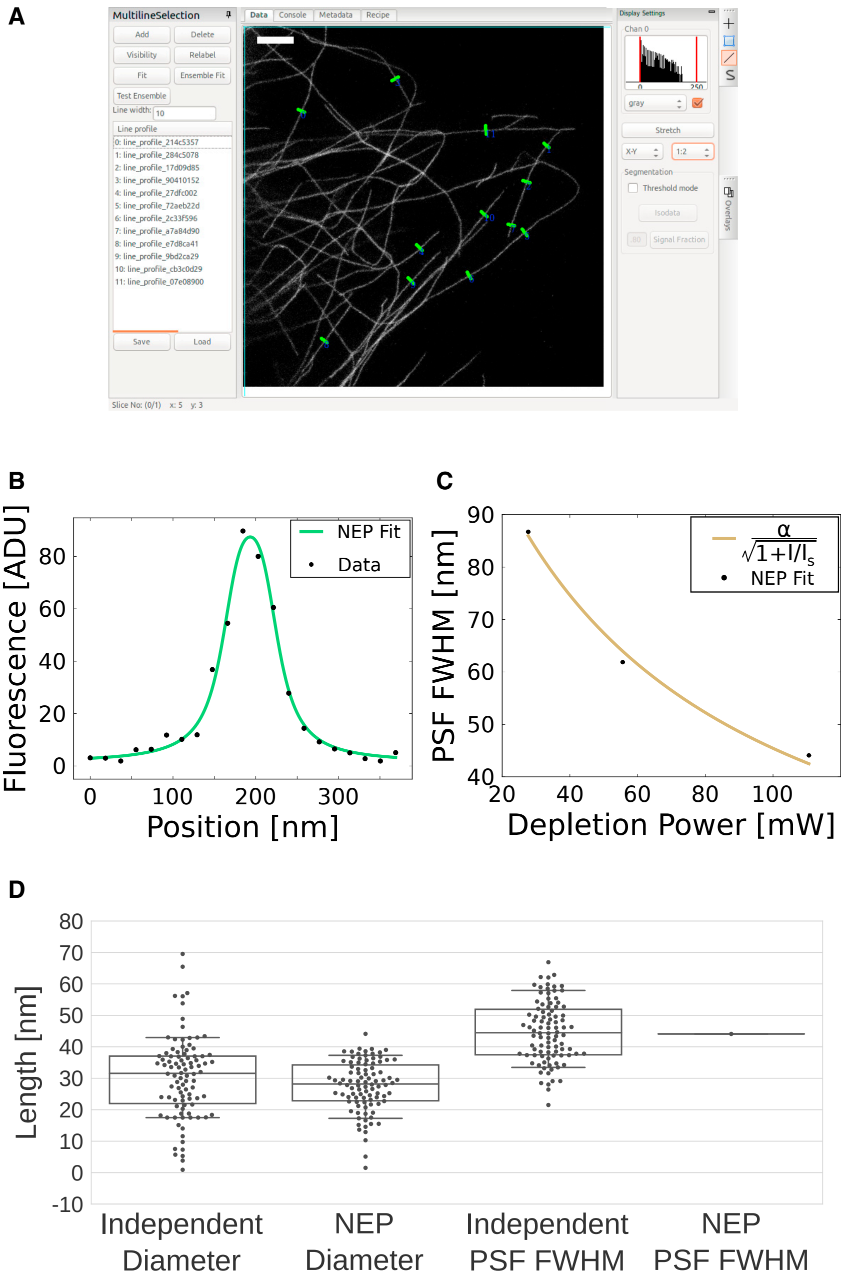

FIGURE 2 (*A*) PYME GUI showing a STED image of immunolabeled microtubules in a COS-7 cell, imaged with 111-mW STED laser power. Green lines show user-selected profiles to be fit. (*B*) Plot of raw data and NEP fit of a microtubule profile from (*A*) are shown. (*C*) Plot of NEP-fitted PSF widths from STED images of microtubules acquired with different STED powers, which scales as expected by theory (n = 74, n = 71, and n = 94 profiles extracted from N = 8, N = 8, and N = 12 images of N = 3, N = 3, and N = 6 cells, acquired at 28, 56, and 111-mW STED laser powers, respectively), are shown. (*D*) Swarm- and box-plots of microtubule diameters and PSF FWHM values determined using NEP fitting, where the PSF is constrained to be the same for all microtubule line profile cross sections, and without NEP fitting (standard least-squares fitting), where the PSF is varied independently for each tubule fit, are shown. To see this figure in color, go online.

diameter and could lead to relative errors of 100% for both values. The minimum number of profiles necessary for robust NEP fitting depends on the fluorophore distribution and the relative PSF size. However, even for cases of low SNR, we found 100 profiles to be sufficient for the fluorophore distributions tested (see Fig. S2). To improve performance in low SNR images, we recommend averaging the line profiles over several pixels width during extraction (as can be done by entering, for example, 10 pixels for the “line width” in the GUI as described in the Supporting Material). This measure, which averages signal along a segment of the tubule, is also effective at reducing any discrepancies between the modeled and true fluorophore distribution that might occur as a result of sparse labeling.

Application to live-cell images

Although the robust PSF measurement in fixed cells by NEP fitting is a substantial improvement over bead calibrations, a large advantage of NEP fitting is that it can be performed on live-cell data for in situ resolution calibration in the most biologically relevant state. We applied ensemble PSF fitting in live-cell STED images of label-filled or

membrane-labeled endoplasmic reticulum (ER) tubules using SNAP-KDEL or SNAP-Sec61β, respectively (Fig. 3, *A–D*). In order to fit the label-filled tubules, we modeled the fluorophore distribution perpendicular to the long axis of the tubule as a filled circle, which projects as $2\sqrt{R^2 - x^2}$, where R is the radius. We then convolved this tubule profile with a Lorentzian to account for the imaging process. For membrane-labeled tubules, we modeled the fluorophore distribution as an annulus, like the antibody-labeled microtubules only with a thinner coat. SNAP-tag (10) is ~4 nm in diameter, and the organic dye itself can be estimated to have a radius of 0.5 nm, assuming they are both globular (11), resulting in a 4.5-nm thick annulus. Fits of ER tubule diameter for various test PSF widths show stronger coupling between the tubule diameter and PSF width for label-filled tubules than membrane-labeled tubules (Fig. 3, *E* and *F*). The PSF width results from the NEP fits were, however, very similar for label-filled and membrane-labeled profiles (45.8 and 43.7 nm FWHM, respectively) as shown in Fig. 3, *G* and *H*.

Notably, the SD for both the label-filled and membrane-labeled ER tubule diameters is fairly large: 30 and 15 nm, respectively (with mean values of 132 and 101 nm). To test whether this variability in tubule diameter is primarily biological in nature or dominated by the SNR-limited fit precision, we simulated tubule profiles of known diameter with similar SNRs, convolved with 50-nm FWHM Lorentzians. The distributions of fitted diameters were narrower than those observed in the live-cell images, with SDs of only 5 nm for the membrane-labeled tubules and 12 nm for the label-filled tubules (Fig. S7). The larger range of label-filled tubule diameters is expected because the fluorophore distribution orthogonal to the long axis of the tubule looks more similar to the PSF than a surface-labeled fluorophore distribution. This is reflected in the live-cell data, where the tubule diameter is more strongly coupled to the PSF width for the label-filled tubules, as shown in the heat-maps of tubule diameter histograms when fit with various fixed PSF widths (Fig. 3, *E* and *F*). Since the standard deviations add in quadrature, we expect roughly 80% of the spread in tubule diameter to be biological in origin.

Discussion

Traditional methods of resolution calibration in STED microscopy are problematic for biological quantification. The NEP fitting method introduced in this article provides a robust and practical alternative to both quantify the performance of a microscope as well as improve feature measurements within the image. Its implementation in a freely downloadable, open source, cross-platform software package allows for rapid adoption by others without requiring mathematical or programming expertise. The principle of ensemble fitting can be readily extended to other fluorescence microscopy modalities (e.g., confocal) by substituting a different functional representation of the PSF when

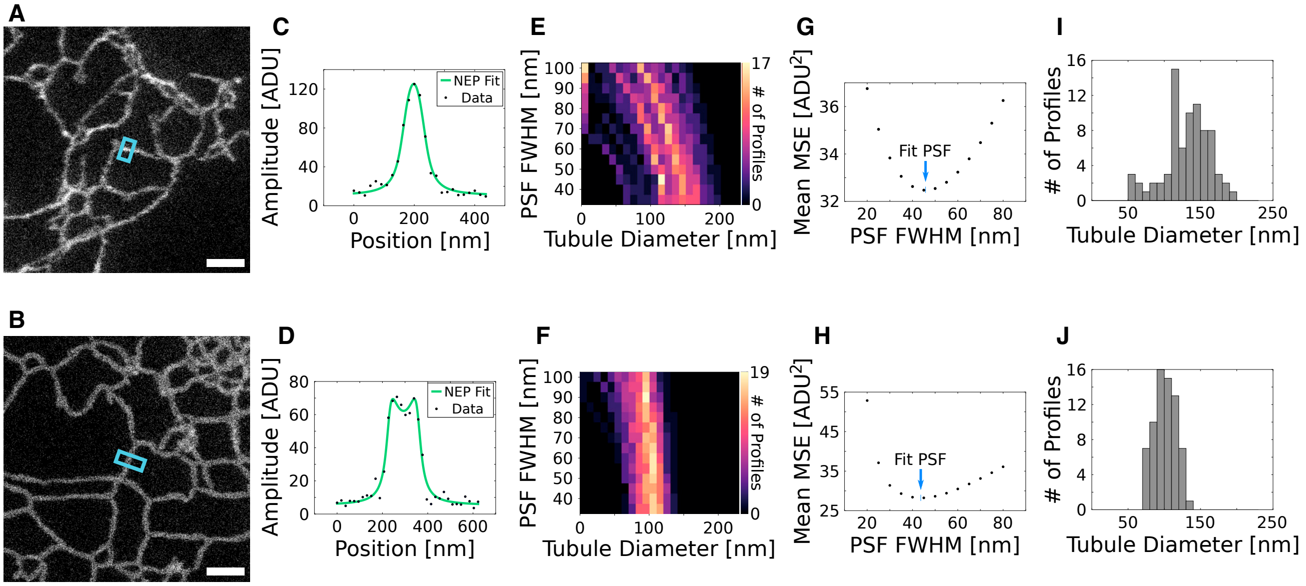

FIGURE 3 (A and B) Live-cell STED images of label-filled ((A) SNAP-KDEL) and membrane-labeled ((B) SNAP-Sec61β) ER. (C and D) Fluorescence line profiles, averaged over 10 pixels along the long axis of the tubule, extracted from (A) and (B), respectively, and fit using NEP fitting. (E and F) Heatmaps showing the coupling between tubule diameter and PSF FWHM for label-filled (E) and membrane-labeled (F) ER when standard least-squares fitting is performed with systematically varied PSF FWHM. n = 77 and n = 69 profiles were extracted from N = 7 and N = 7 STED images of N = 4 and N = 2 cells for (E) and (F), respectively. (G and H) Mean MSE values for fits shown in (E) and (F), respectively. The blue arrow indicates the PSF FWHM found by performing NEP fitting on the same tubule line profiles. (I and J) Label-filled (I) and membrane-labeled (J) ER tubule diameters fit using NEP fitting (45.8-nm PSF FWHM for SNAP-KDEL, 43.7-nm PSF FWHM for SNAP-Sec61β). The mean and standard deviations were 132 ± 30 and 101 ± 15 nm for (I) and (J), respectively. Scale bars, 1 μm. ADU, analog-digital units. To see this figure in color, go online.

producing the model function for fitting; the only requirement is that the labeling geometry of the structure is known. This known geometry is not limited to tubules and can be extended to fit objects like beads or vesicles, which would be useful for cell-trafficking studies. The accurate measure of PSF width afforded by NEP fitting can be used to quantify microscope performance under various conditions, refine models of organelle morphology, and remove uncertainty in parameter selection for deconvolution or other image enhancement algorithms.

Software availability

All line profiles were drawn, extracted, and fit using the open source PYME and the NEP fitting plug-in, which are both freely available (12,13). Supplemental data are provided alongside this article for readers to test the program.

SUPPORTING MATERIAL

Supporting Materials and Methods, seven figures, and one data file are available at http://www.biophysj.org/biophysj/supplemental/S0006-3495(18)30915-9.

AUTHOR CONTRIBUTIONS

A.E.S.B., D.B., and J.B. designed the research. A.E.S.B. developed the technique and model functions and performed simulations and data analysis. A.E.S.B., L.K.S., M.G., D.B., and J.B. wrote the manuscript. L.K.S. optimized and performed all imaging and live-cell sample preparations. A.E.S.B., M.G., and D.B. developed the plug-in for PYME. PYME has been developed by D.B.

ACKNOWLEDGMENTS

We thank Phylicia Kidd and Mark Lessard for providing biological test samples, and Zach Marin for helpful discussions. J.B. discloses significant financial interest in Bruker Corp. and Hamamatsu Photonics.

This work is supported by the G. Harold & Leila Y. Mathers Foundation, the Wellcome Trust (095927/A/11/Z and 203285/B/16/Z), the Yale Diabetes Research Center (NIH P30 DK045735), and National Institutes of Health grant S10 OD020142 for imaging resources (for the Leica TCS SP8 STED 3X microscope). A.E.S.B. acknowledges support by a National Institutes of Health training grant (T32 GM008283).

SUPPORTING CITATIONS

References (14–19) appear in the Supporting Material.

REFERENCES

1. Weber, K., P. C. Rathke, and M. Osborn. 1978. Cytoplasmic microtubular images in glutaraldehyde-fixed tissue culture cells by electron microscopy and by immunofluorescence microscopy. *Proc. Natl. Acad. Sci. USA*. 75:1820–1824.
2. Vicidomini, G., A. Schönle, …, S. W. Hell. 2013. STED nanoscopy with time-gated detection: theoretical and experimental aspects. *PLoS One*. 8:e54421.
3. Persson, F., P. Bingen, …, S. W. Hell. 2011. Fluorescence nanoscopy of single DNA molecules by using stimulated emission depletion (STED). *Angew. Chem. Int.Engl*. 50:5581–5583.
4. Nieuwenhuizen, R. P., K. A. Lidke, …, B. Rieger. 2013. Measuring image resolution in optical nanoscopy. *Nat. Methods*. 10:557–562.
5. Banterle, N., K. H. Bui, …, M. Beck. 2013. Fourier ring correlation as a resolution criterion for super-resolution microscopy. *J. Struct. Biol*. 183:363–367.
6. Tortarolo, G., M. Castello, …, G. Vicidomini. 2018. Evaluating image resolution in stimulated emission depletion microscopy. *Optica*. 5:32–35.
7. Baddeley, D., Y. Weiland, …, C. Cremer. 2010. Model based precision structural measurements on barely resolved objects. *J. Microsc*. 237:70–78.
8. Meyer, L., D. Wildanger, …, S. W. Hell. 2008. Dual-color STED microscopy at 30-nm focal-plane resolution. *Small*. 4:1095–1100.
9. Hell, S. W. 2007. Far-field optical nanoscopy. *Science*. 316:1153–1158.
10. Keppler, A., S. Gendreizig, …, K. Johnsson. 2003. A general method for the covalent labeling of fusion proteins with small molecules in vivo. *Nat. Biotechnol*. 21:86–89.
11. Erickson, H. P. 2009. Size and shape of protein molecules at the nanometer level determined by sedimentation, gel filtration, and electron microscopy. *Biol. Proced. Online*. 11:32–51.
12. Baddeley, D. Python Microscopy Environment. www.python-microscopy.org.
13. Barentine, A. E. S., M. Graff, and D. Baddeley. Code Repository: Nested-Loop Ensemble Fitting. www.github.com/bewersdorflab/nep-fitting.
14. Harke, B., J. Keller, …, S. W. Hell. 2008. Resolution scaling in STED microscopy. *Opt. Express*. 16:4154–4162.
15. Riley, K., M. Hobson, and S. Bence. 2006. Mathematical Methods for Physics and Engineering, Third Edition. Cambridge University Press, Cambridge, UK.
16. Weisstein, E. W. Fourier Series–Semicircle. From MathWorld—A Wolfram Web Resource. http://mathworld.wolfram.com/FourierSeriesSemicircle.html.
17. Huang, F., G. Sirinakis, …, J. Bewersdorf. 2016. Ultra-high resolution 3D imaging of whole cells. *Cell*. 166:1028–1040.
18. Schroeder, L. K., A. E. S. Barentine, …, S. Bahmanyar. 2017. Nanoscale size holes in ER sheets provide an alternative to tubules for highly-curved membranes. *bioRxiv* https://doi.org/10.1101/191536.
19. Bottanelli, F., E. B. Kromann, …, J. Bewersdorf. 2016. Two-colour live-cell nanoscale imaging of intracellular targets. *Nat. Commun*. 7:10778.

Biophysical Journal, Volume *115*

Supplemental Information

Simultaneously Measuring Image Features and Resolution in Live-Cell STED Images

Andrew E.S. Barentine, Lena K. Schroeder, Michael Graff, David Baddeley, and Joerg Bewersdorf

Supplementary Figures

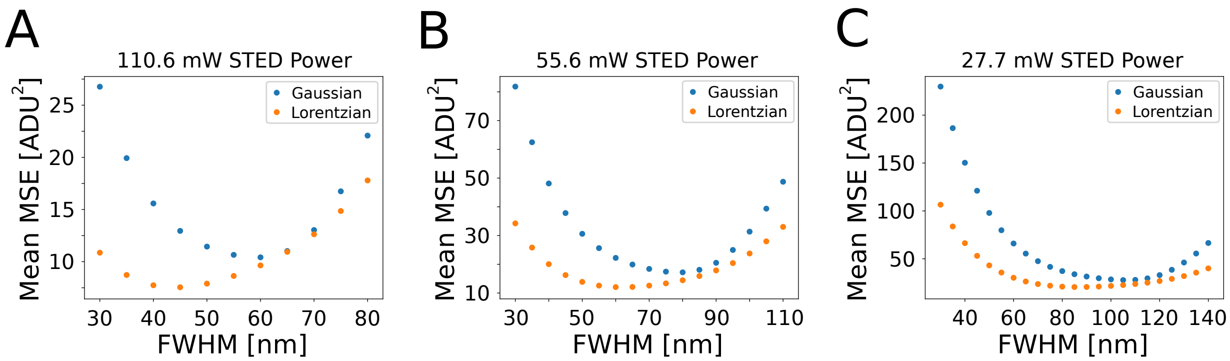

Figure S1: Comparison of Lorentzian and Gaussian PSF models applied to the microtubule data shown in figure 2. (**A**, **B**, **C**) MSE averaged over all profiles of NEP fit as a function of PSF FWHM when the NEP fitting model function is derived for a Gaussian (blue) or Lorentzian (orange) PSF model. STED laser powers were 110.6 mW, 55.6 mW, and 27.7 mW for (A), (B) and (C), respectively. The Lorentzian is a better model for our PSF as shown by the average MSE being consistently smaller compared to the Gaussian model for all tested STED laser powers. The difference is most notable (about a factor of 2) for 110.6 mW, which matches that used in our live-cell imaging. Approximating the depletion donut as a parabola with pulsed excitation, pulsed depletion and time-gated detection should produce a Gaussian-shaped effective PSF (1). Our experimental observation of a more Lorentzian-shaped effective PSF might be explained by the preferential excitation and depletion of certain dipole orientations of the fluorophore, which can have a significant impact on PSF shape.

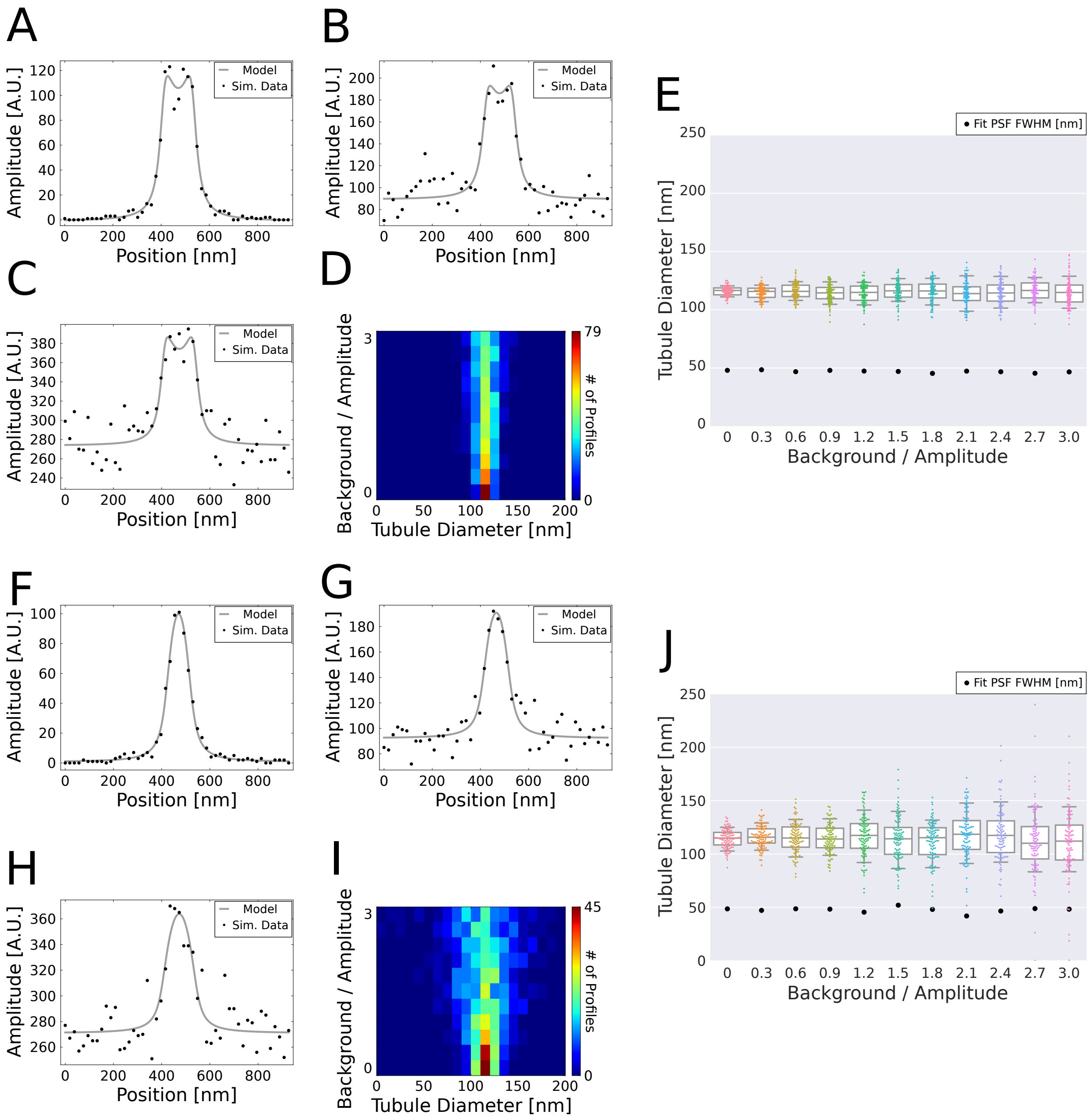

Figure S2: Effect of noise on ER tubule diameter and PSF fitting. (**A**, **B**, **C**) Simulated surface-labeled ER tubules with background to amplitude ratios of 0 (A), 0.9 (B), and 2.7 (C). (**D**) Heatmap of fitted diameters from NEP fits of simulated surface-labeled ER tubule profiles with varied background to amplitude ratios, and therefore varied signal-to-noise ratios. (**E**) Fitted diameters from NEP fits of simulated surface-labeled ER tubules with different background to amplitude ratios. The NEP-fit PSF width is plotted in black. N=100 simulated tubules for each ratio. (**F**, **G**, **H**) Simulated label-filled ER tubules with background to amplitude ratios of 0 (F), 0.9 (G), and 2.7 (H). (**I**) Heatmap of fitted diameters from NEP fits of simulated profiles of label-filled ER tubules with varied background to amplitude ratios, and therefore signal-to-noise ratios. (**J**) Fitted diameters from NEP fits of simulated label-filled ER tubules with different background to amplitude ratios. The NEP-fit PSF width is also plotted in black. N=100 simulated tubules for each ratio. The whiskers of boxplots represent the 10th and 90th percentiles of each distribution, the colored boxes cover the interquartile range, and the center line in each box denotes the median.

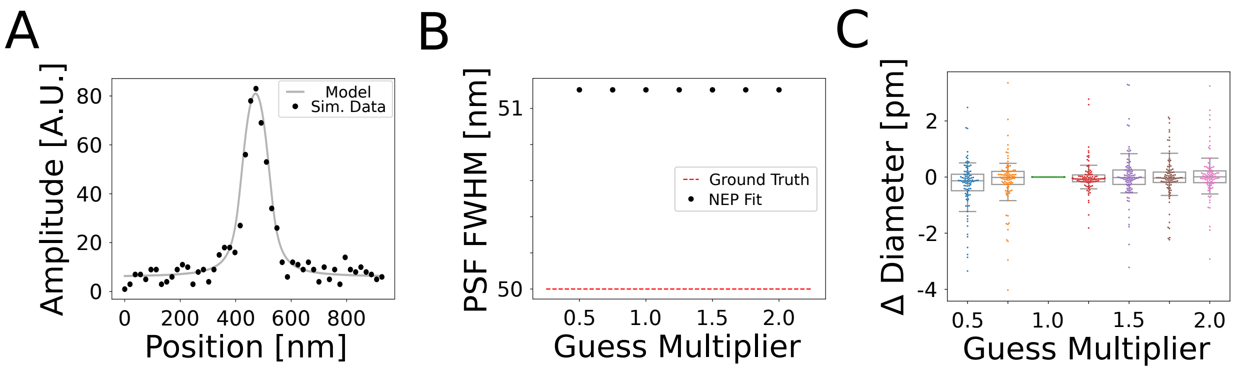

Figure S3: Both estimated tubule diameters and PSF FWHMs are independant of initial parameter guesses. (**A**) Example of a simulated 115 nm-diameter label-filled ER tubule convolved with a 50 nm FWHM Lorentzian PSF, with added Poisson noise. (**B**) NEP-fit PSF FWHM as a function of initial guesses for the fit parameters (including the PSF width). The initial guesses for fit parameters are calculated automatically, and then multiplied by a guess multiplier (abscissa) with the exception of the center position guess, which was shifted by one PSF FWHM (50 nm). The NEP-fit PSF width is plotted in black, the ground truth is represented by the dashed red line. While there is a small systematic error in the 1-nm range, the NEP-fit PSF width results are independent of the initial guesses for fit parameters. (**C**) The NEP-fit tubule diameters resulting from a guess multiplier of 1 (i.e. default automated guesses with the center position shifted by 50 nm) were subtracted from the NEP-fit tubule diameters resulting from the altered initial guesses. Notably, the effect of altering the initial guesses by a factor of two in either direction is very small, resulting in changes on the picometer-range, while the standard deviation of tubule diameters is roughly 10,000 times larger at 12.26 nm. N=100 simulated tubules for each guess multiplier. The whiskers of boxplots in (*B*) and (*C*) represent the 10th and 90th percentiles of each distribution, the boxes cover the interquartile range, and the center line in each box denotes the median.

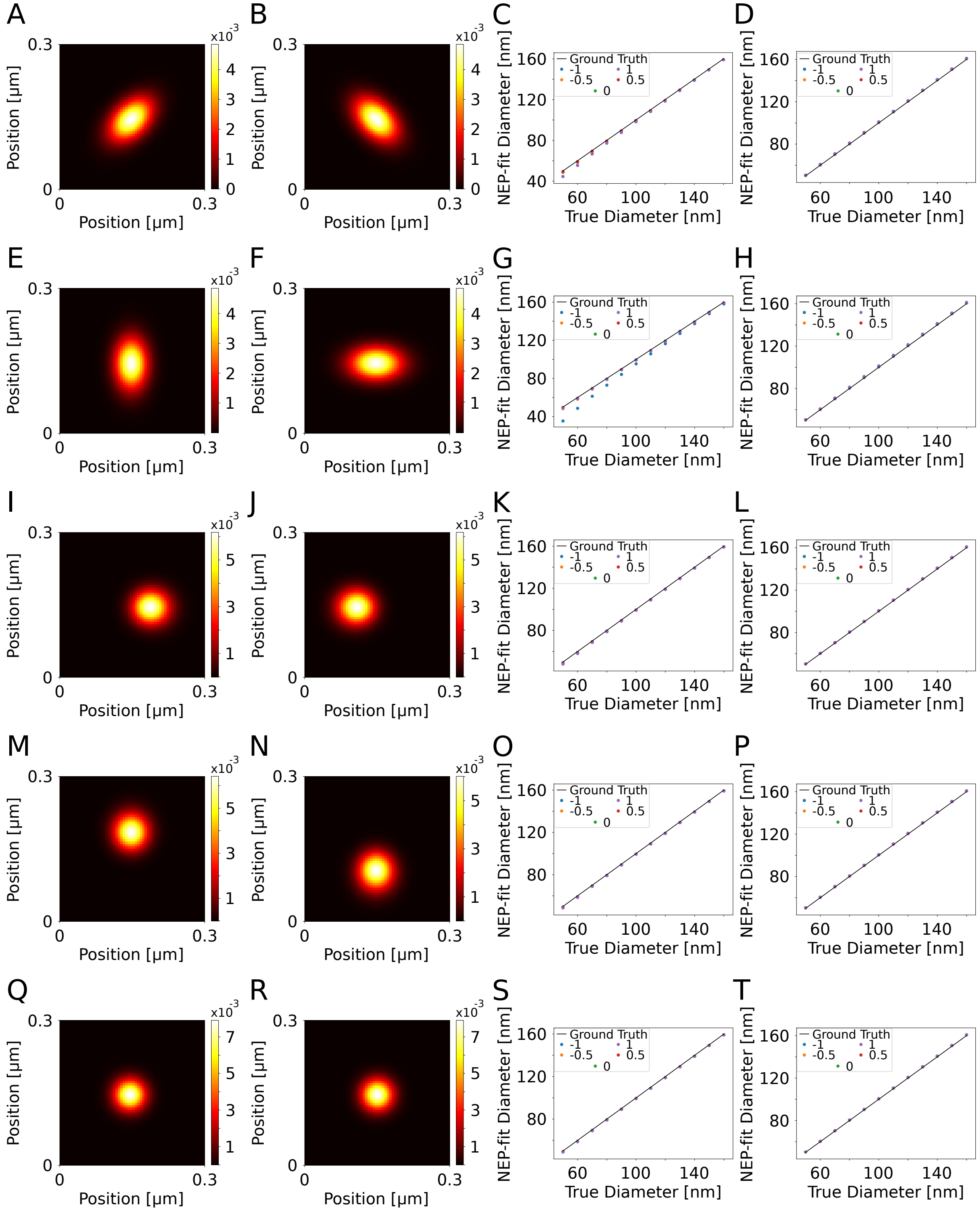

Figure S4: NEP-fitted tubule diameter measurements are robust to ±1 radian Zernike aberrations. A Gaussian (rather than Lorentzian) NEP-fitting model was used as the simulated steady-state STED process has a Gaussian-shaped PSF (2), which differs from our pulsed excitation, pulsed emission, time-gated detection physical implementation (see Methods Section below). The effective STED PSF is shown for ±1 radian aberrations for the 4th-8th Zernike modes, which are x astigmatism (**A**, **B**), y astigmatism (**E**, **F**), x coma (**I**, **J**), y coma (**M**, **N**), and spherical (**Q**, **R**). To the right of these PSFs are plots of the NEP-fitted diameters vs. the ground-truth diameters after fitting simulated label-filled (**C**, **G**, **K**, **O**, **S**) and surface-labeled (**D**, **H**, **L**, **P**, **T**) tubules. The color of the plotted points represents the magnitude of the respective aberration, in radians.

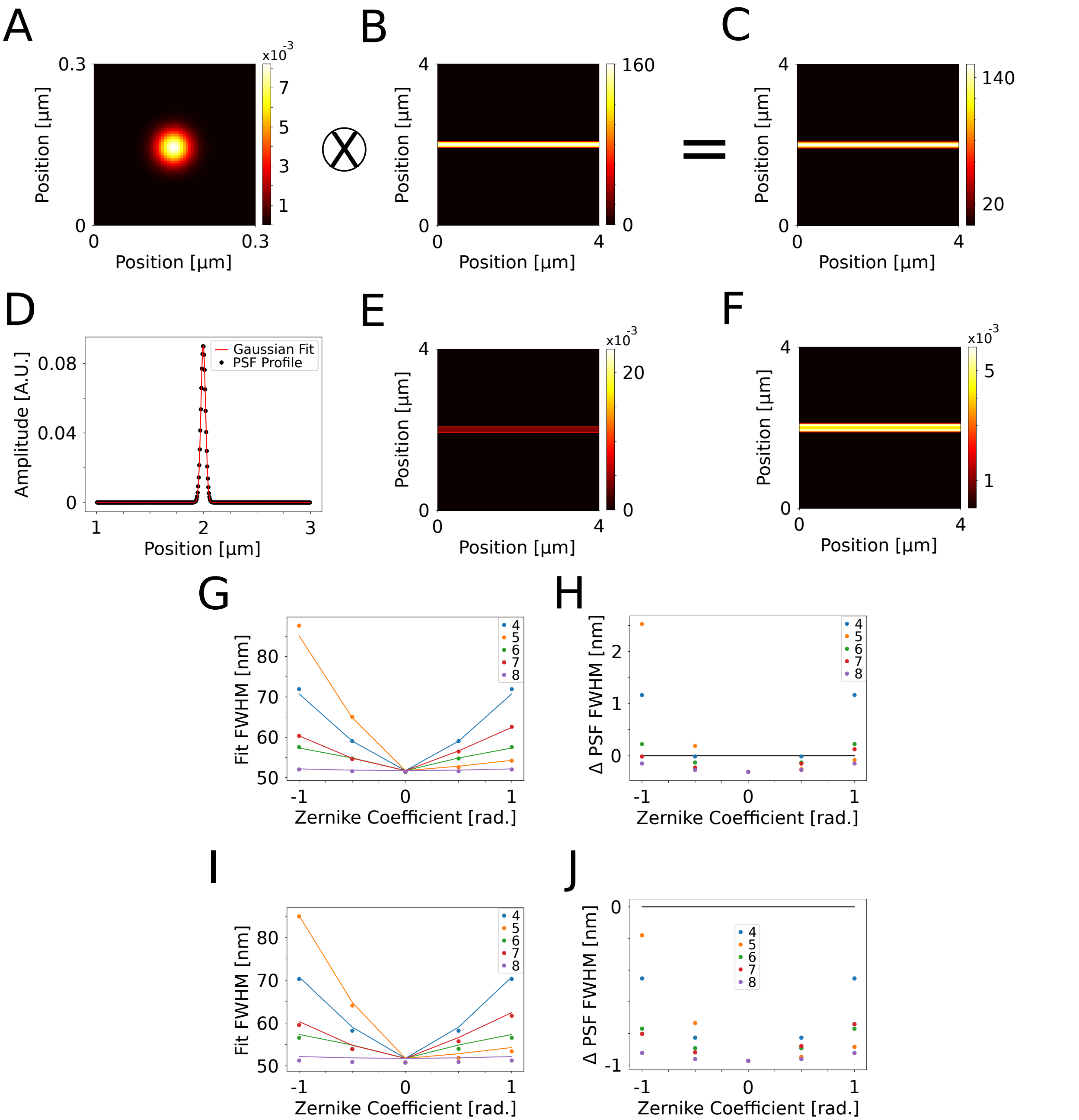

Figure S5: NEP-fitted PSF FWHM estimates are robust to ±1 radian Zernike aberrations. (**A**) Zoom in of unaberrated steady-state STED effective PSF (Gaussian profile (2), see Methods Section below), whose profile is fit in (**D**). (**B**, **E**) Fluorophore distributions for 160-nm diameter label-filled (B) and surface-labeled (E) tubules. (**C**, **F**) Tubules from (B) and (E), respectively, after being convolved with the unaberrated PSF shown in (A). (**G**, **I**) NEP-fitted PSF FWHM as a function of Zernike-mode aberration coefficient for 5 different Zernike modes (colored points) performed on label-filled (G) and surface-labeled (I) tubules ranging from 50 - 160 nm diameter. The lines represent the effective ground-truth, which is determined by fitting a Gaussian to a profile of the PSF itself, as shown in (D). (**H**, **J**) NEP-fitted PSF FWHM minus the ground-truth PSF FWHM as a function of aberration strength for both label-filled (H) and surface-labeled (J) tubules ranging from 50 - 160 nm diameter. Perfect agreement is denoted with a black line at ΔPSF FWHM = 0. Notably, agreement is within 3 nm and 1 nm for label-filled and surface-labeled tubules, respectively. As expected, the agreement is better for the surface-labeled tubules. The color of each point in (G, H, I J) represent the 4th-8th Zernike-modes describing the aberrations which correspond to x astigmatism (4), y astigmatism (5), x coma (6), y coma (7), and spherical (8).

STEDTubule_SurfaceAntibody fits
for /Users/Andrew/Downloads/NEP-fitting_supplementary/roomTemp_anti-tubulin_COS7.tif

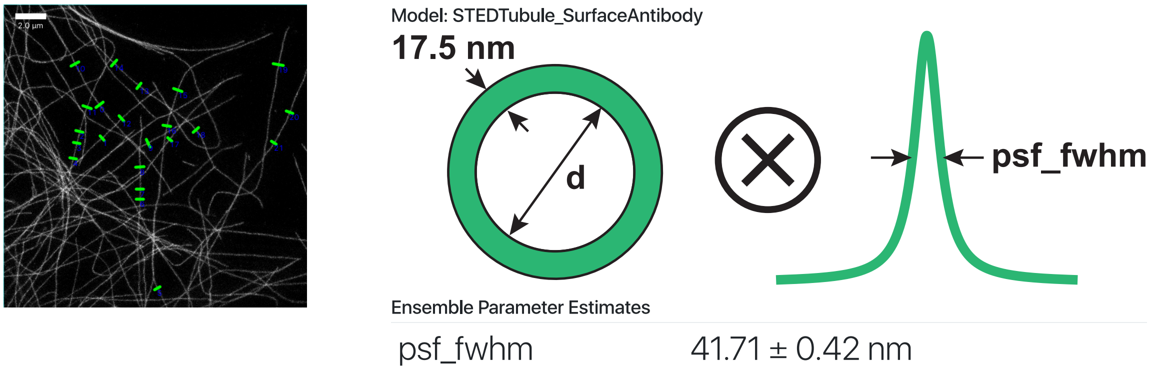

Ensemble Parameter Estimates

psf_fwhm	41.71 ± 0.42 nm

Individual fit parameters

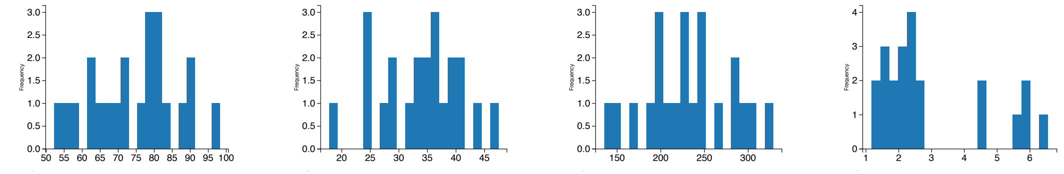

Index	amplitude	diameter	center	background
0	72.71 ± 1.96	47.44 ± 2.50	287.86 ± 1.00	1.45 ± 0.60
1	88.58 ± 2.53	17.79 ± 2.91	145.31 ± 0.75	2.44 ± 0.71
2	70.54 ± 2.61	39.21 ± 3.38	242.74 ± 1.26	2.15 ± 0.85
3	80.53 ± 1.45	28.01 ± 1.71	220.36 ± 0.54	2.44 ± 0.44

Figure S6: A (cropped) NEP-fitting report. 22 profiles were selected from the provided anti-tubulin image (see supplementary data) and then NEP-fitting was performed using the Lorentzian-convolved antibody-coated tubule model, as shown. Reports are automatically generated when calling fitting, NEP-fitting, or test ensemble values functions from the GUI. Reports are saved as html pages and can be opened with standard web browsers.

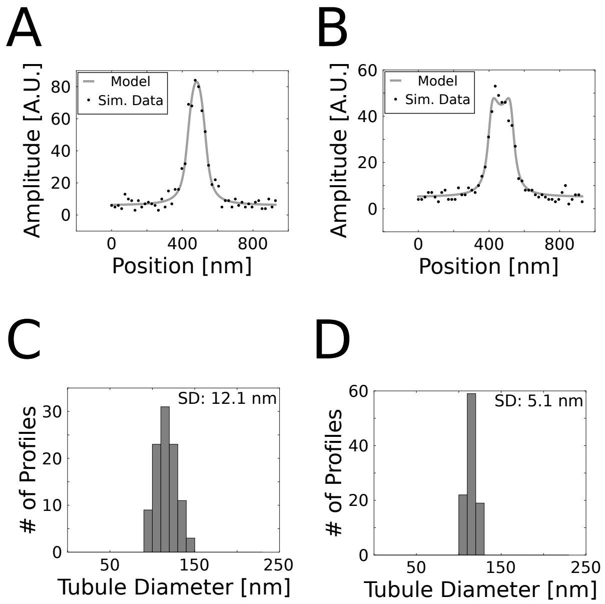

Figure S7: Variance of diameter fits for simulated 115-nm diameter tubules. (**A**, **B**) Plots of simulated profiles of a label-filled (A) and surface-labeled (B) tubule with 115-nm diameter, convolved with a 50-nm FWHM Lorentzian PSF, the expected PSF of our STED microscope. (**C**, **D**) Histograms of fitted tubule diameters for simulated 115-nm diameter label-filled (C) and surface-labeled (D) tubules. NEP fit diameters for the label-filled tubule profiles had a mean of 116 $\pm$ 12 nm (mean $\pm$ SD), and the NEP fit PSF width was 47.5 nm for these profiles. NEP fit diameters for the surface-labeled tubule profiles had a mean of 115 $\pm$ 5 nm (mean $\pm$ SD), and the NEP fit PSF width was 49.3 nm for these profiles. N=100 simulated profiles for both label-filled and surface-labeled models.

Materials and Methods

Tubule Model Functions

The model functions used in ensemble PSF fitting were derived by taking the projection of the fluorescence labeling geometry onto the xy-plane and then convolving this projection with a model of the microscope PSF. In this work we chose a Lorentzian PSF model since it resembled our experimental PSF profiles well (see Fig. S1). Derivation of the functions used was carried out in Mathematica (Wolfram Research, Champaign, IL).

Principle

Inherent to the imaging process is the convolution of the Point-Spread Function (PSF) with the structure being imaged.

$$I(x, y, z) = h(x, y, z) \otimes s(x, y, z) \tag{1}$$

where I is the resulting image, h is the PSF, and s is the fluorophore distribution. We only consider tubule cross sections because the profile of the tubule is uniform along its long-axis (y), and can therefore write the convolution as

$$I_{\text{cs}}(x, z) = \int_{-\infty}^{\infty} \int_{-\infty}^{\infty} h(\tau_1, \tau_2) s(x - \tau_1, z - \tau_2) d\tau_1 d\tau_2, \tag{2}$$

where I_{cs} is the cross section of the 3D image of the tubule.

For standard STED microscopy, the full-width at half-maximum (FWHM) of the PSF along the axial dimension is significantly larger than the tubular structures we consider, such that the convolution along the axial dimension reduces to a sum. We can now write

$$P(x) = \int_{-\infty}^{\infty} h(\tau) p(x - \tau) d\tau, \tag{3}$$

where P is the line profile cross section, p is the projection of s (summed over z), and h is the 1D lateral profile of the PSF.

Annulus fluorophore distribution

The cross section of a surface-labeled tubule can be taken to be an annulus, where we assume that the fluorophores are uniformly distributed between the inner and the outer radius. Again, due to the large axial FWHM of the STED PSF, the convolution along the axial dimension reduces to a sum. We calculate this sum by considering half of an annulus, and subtracting the z position of the inner radius edge, $z_i(x)$, from the outer radius edge, $z_o(x)$,

$$z_i(x) = r \sin(\theta_i), \tag{4}$$

and

$$z_o(x) = R \sin(\theta_o), \tag{5}$$

respectively, where R is the outer radius of the annulus, r is the inner radius, $\theta_o = \arccos\left(\frac{x}{R}\right)$, and $\theta_i = \arccos\left(\frac{x}{r}\right)$. Note that outside of the inner or outer radius, respectively, $z_i(x)$ and $z_o(x)$ switch from purely real-valued to purely imaginary, which can be easily accounted for later, since the PSF they will be multiplied by in the convolution is purely real-valued. We can now write the projection of the annulus simply as

$$p_{\text{annulus}}(x) = \frac{2\left(z_o(x) - z_i(x)\right)}{\pi\left(R^2 - r^2\right)}, \tag{6}$$

where the factor of two accounts for the top and bottom halves of the annulus, and we have sum-normalized $p_{\text{annulus}}(x)$. The Lorentzian function in 1D is given by

$$L(x) = \frac{1}{2\pi} \frac{\gamma}{x^2 + \left(\frac{\gamma}{2}\right)^2}, \tag{7}$$

where γ is the FWHM. Substituting $L(x)$ for the 1D PSF, we can determine the line profile intensity of an annulus structure imaged with a Lorentzian PSF by evaluating

$$P_{\text{annulus}}(x) = \int_{-\infty}^{\infty} L(\tau) p_{\text{annulus}}(x-\tau) d\tau. \tag{8}$$

The integral in equation 8 can be evaluated using Cauchy's residue theorem,

$$\oint_C f(\tau) d\tau = 2\pi i \sum_j \text{Res}_j, \tag{9}$$

where C is a closed contour and Res_j are the residues of the poles within C. Our integrand, $L(\tau)p_{\text{annulus}}(x-\tau)$, has simple poles at $\tau = \pm i\gamma/2$. We therefore choose C to be the semicircular contour shown in figure S8, where we integrate along the real axis from $\tau = -\infty$ to $\tau = \infty$, and then around the arc of $\tau = \lim_{R' \to \infty} R' e^{i\theta}$ from $\theta = 0$ to $\theta = \pi$, which encloses the pole at $\tau = i\gamma/2$.

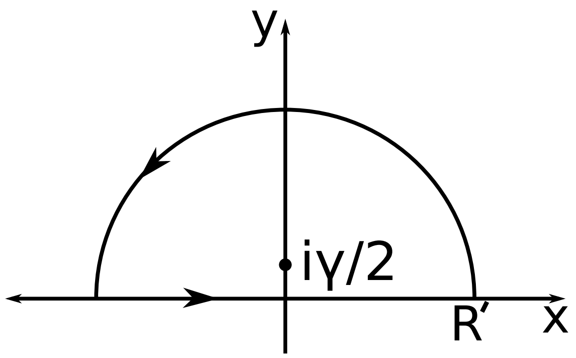

Figure S8: The contour C chosen to integrate over in the application of equation 9.

Equation 9 can now be expanded to

$$\oint_C L(\tau) p_{\text{annulus}}(x-\tau) d\tau = 2\pi i \sum_j \text{Res}_j \tag{10}$$

$$= \int_{-\infty}^{\infty} L(\tau) p_{\text{annulus}}(x-\tau) d\tau + \lim_{R' \to \infty} \int_0^{\pi} L(R'e^{i\theta}) p_{\text{annulus}}(x - R'e^{i\theta}) d\theta \tag{11}$$

$$= P_{\text{annulus}}(x) + \int_0^{\pi} L(R'e^{i\theta}) p_{\text{annulus}}(x - R'e^{i\theta}) d\theta \tag{12}$$

$$= 2\pi i \text{Res}_{\tau = i\gamma/2}. \tag{13}$$

Conveniently, $\lim_{R' \to \infty} L(R'e^{i\theta}) p_{\text{annulus}}(x - R'e^{i\theta}) = 0$, so $P_{\text{annulus}}(x) = 2\pi i \text{Res}_{\tau = i\gamma/2}$. The residue $\text{Res}_\tau = i\gamma/2$ can be calculated as $\left.\frac{g(\tau)}{h'(\tau)}\right|_{\tau = i\gamma/2}$ where g is the numerator of $L(\tau)p_{\text{annulus}}(x-\tau)$, and h' is the derivative of the denominator with respect to τ (3). This yields

$$P_{\text{annulus}}(x) = \frac{1}{\pi (r^2 - R^2)} \left[\sqrt[4]{16x^2\gamma^2 + (4r^2 - 4x^2 + \gamma^2)^2} \cos\left(\frac{1}{2} \arctan\left(4r^2 - 4x^2 + \gamma^2, 4x\gamma\right)\right) \right. \tag{14}$$

$$\left. - \sqrt[4]{16x^2\gamma^2 + (4R^2 - 4x^2 + \gamma^2)^2} \cos\left(\frac{1}{2} \arctan\left(4R^2 - 4x^2 + \gamma^2, 4x\gamma\right)\right) \right], \tag{15}$$

where $\arctan(x, y)$ is the quadrant-respecting inverse tangent function (referred to as $\arctan 2$ in several programming languages). Note that we only consider the real part of $P_{\text{annulus}}(x)$, as the imaginary component was introduced by not defining $p_{\text{annulus}}(x)$ as a piecewise function. We verified this approach by performing the convolution in Mathematica using a piecewise-defined $p_{\text{annulus}}(x)$, which achieved the same result.

Label-filled Model Function

In order to derive a label-filled model function, which can be used to model a lumen-labeled ER tubule, we have two options. The first approach would be to follow the same steps as above using the projection of a circle, which is a semi-circle

$$p_{\text{circle}}(x) = \sqrt{(R-x)(R+x)}, \tag{16}$$

where R is the radius. However, we can also simply take the limit as the inner radius of the annulus model function goes to zero, which yields

$$P_{\text{circle}}(x) = \int_{-\infty}^{\infty} L(\tau) p_{\text{circle}}(x-\tau) d\tau \tag{17}$$

$$= \lim_{r \to 0} P_{\text{annulus}}(x) \tag{18}$$

$$= \frac{1}{\pi R^2} \left[-\sqrt[4]{(4x^2+\gamma^2)^2} \cos\left(\frac{1}{2}\arctan\left(\gamma^2 - 4x^2, 4x\gamma\right)\right) \right. \tag{19}$$

$$\left. + \sqrt[4]{16x^2\gamma^2 + (4R^2 - 4x^2 + \gamma^2)^2} \cos\left(\frac{1}{2}\arctan\left(4R^2 - 4x^2 + \gamma^2, 4x\gamma\right)\right)\right]. \tag{20}$$

Gaussian-Convolved Model Functions

Confocal PSFs and PSFs for some STED modalities are better approximated by a Gaussian rather than a Lorentzian (1). While this is not the case for our experimental data, our steady-state STED simulations yield a Gaussian PSF (see Fig. S5D), as expected (2).

The (normalized) Gaussian function in 1D is given by

$$G(x) = \frac{\exp\left(-\frac{x^2}{2\sigma^2}\right)}{\sqrt{2\pi}\sigma}. \tag{21}$$

We can then write the model function for a label-filled tubule convolved with a Gaussian PSF as

$$\tilde{P}_{\text{circle}}(x) = \int_{-\infty}^{\infty} G(x-\tau) p_{\text{circle}}(\tau) d\tau, \tag{22}$$

where the tilde has been introduced to denote the Gaussian PSF model. We expanded the semicircle with a Fourier cosine series, which is given by

$$p_{\text{circle}}(x) = R\left(\frac{\pi}{2} + \sum_n \frac{(-1)^n J_1(n\pi)\cos\left(\frac{\pi n(x-R)}{R}\right)}{n}\right), \tag{23}$$

where J_1 is a Bessel function of the first kind (4) and we have not yet made any approximations.

Fortunately, convolutions are linear operators, so we can split up our convolution integral into an integral for each term in the expansion,

$$\tilde{P}_{\text{circle}}(x, R) = \int_{-R}^{R} G(x-\tau) R\left(\frac{\pi}{2} + \sum_n \frac{(-1)^n J_1(n\pi)\cos\left(\frac{\pi n(\tau-R)}{R}\right)}{n}\right) d\tau \tag{24}$$

$$= \int_{-R}^{R} G(x-\tau)\frac{\pi R}{2} d\tau + \sum_n \left[\int_{-R}^{R} G(x-\tau)\frac{R(-1)^n J_1(n\pi)\cos\left(\frac{\pi n(\tau-R)}{R}\right)}{n} d\tau\right] \tag{25}$$

$$= \Lambda_\circ + \sum_n \Lambda_n, \tag{26}$$

where

$$\Lambda_\circ = \frac{1}{4}\pi R\left(\text{erf}\left(\frac{R+x}{\sqrt{2}\sigma}\right) - \text{erf}\left(\frac{x-R}{\sqrt{2}\sigma}\right)\right), \tag{27}$$

and

$$\Lambda_n = \frac{R(-1)^n J_1(n\pi) e^{-\frac{\pi n\left(\pi n\sigma^2+2iR(R+x)\right)}{2R^2}}}{4n} \times \tag{28}$$
$$\left[\operatorname{erf}\left(\frac{\lambda + R^2 - Rx}{\sqrt{2}R\sigma}\right) + \operatorname{erf}\left(\frac{R(R+x)-\lambda}{\sqrt{2}R\sigma}\right) + e^{\frac{2i\pi nx}{R}}\left(\operatorname{erf}\left(\frac{-\lambda + R^2 - Rx}{\sqrt{2}R\sigma}\right) + \operatorname{erf}\left(\frac{R(R+x)+\lambda}{\sqrt{2}R\sigma}\right)\right)\right].$$

Where erf is the Gauss error function and we have introduced $\lambda = i\pi n\sigma^2$. Again leveraging the linear nature of convolutions, we can easily define the Gaussian-convolved annulus model function by subtracting two Gaussian-convolved circles where the circles have different radii.

$$\tilde{P}_{\text{annulus}}(x) = \tilde{P}_{\text{circle}}(x, R) - \tilde{P}_{\text{circle}}(x, r), \tag{29}$$

where $r < R$.

We then approximate $\tilde{P}_{\text{circ}}(x)$, and $\tilde{P}_{\text{annulus}}(x)$ by truncating each series after $n = 5$. Our approximation matches the numerical convolution quite well, but is advantageous as it avoids discrete sampling issues.

Deriving New Model Functions

Similar model functions can be derived for other target structures and PSF model functions, with the caveat that they might not be as simple as the tubule models above. Tubules and other linear structures represent an easy class of structure to model because the cross-section is relatively uniform along the long-axis of the tubule, allowing the convolution to be ignored along that direction, and the model function to be generated only considering a single dimension. Other geometries do not allow this reduction in dimensionality, and the convolution integrals must be performed in 2 or 3D. This makes analytic model functions for STED microscopy, in particular, difficult, as 2D Lorentzians cannot be analytically normalized. In the absence of a closed analytic form, NEP fitting can be performed using numeric model functions albeit with significantly poorer computational speed.

Microtubule Simulations

Microtubule line profiles were simulated using a Lorentzian-convolved annulus model function, where the annulus had an inner diameter of 25 nm and outer diameter of 60 nm to account for a dense primary- and secondary-antibody coat (5). The center position of each microtubule was randomly varied at the sub-pixel level to avoid aliasing, and the values generated from the model were then used as expectation values in generating and sampling Poisson distributions to add shot noise to the model. The amplitude and background levels were chosen such that the signal-to-noise ratio is comparable to our fixed-cell microtubule images. The FWHM of the Lorentzian-shaped PSF was varied between 20 and 100 nm, and 50 microtubule line profiles were simulated with each PSF width.

ER Tubule Simulations

ER tubule line profiles were simulated using both label-filled and surface-labeled tubule model functions, where the model function describes the expected shape of a line profile drawn perpendicular to the long axis of a straight region of tubule. The fluorophore distribution for the surface label was taken to be an annulus of 115 nm inner diameter projected onto a line, with outer diameter 124 nm, where the 4.5 nm thickness is to account for the SNAP-tag and organic dye molecule, which were both assumed to be globular in estimating their diameters (6). The label-filled fluorophore distribution was modeled as a circle of 115 nm diameter projected onto a line. The fluorophore distributions for each model were convolved with a Lorentzian of 50 nm FWHM to emulate the microscope resolution. First, 100 profiles of each model were simulated, with their center positions randomly varied at the sub-pixel level to avoid aliasing. The intensity values generated by each model were used as expected values in generating and sampling Poisson distributions to add shot noise levels comparable to the SNAP-KDEL and SNAP-Sec61β live-cell images contained in this work. Ensemble fitting was then performed on the tubules corresponding to each model, and the fitted diameters were plotted in histograms (Fig. S1). Second, we simulated profiles at various signal-to-noise levels. The profiles were

generated in the same way, except that the pre-shot-noise background was varied from 0 to 300, while the amplitude was kept constant at 100. Due to the nature of Poisson statistics, this generates tubules with substantially different noise levels. 100 line profiles were simulated at each noise level.

Fitting

Fitting was performed in PYME using the Scipy package, specifically the Levenberg-Marquardt and Nelder-Mead minimization algorithms. For standard, non-NEP fitting, profile fits were performed with the Scipy package Levenberg-Marquardt implementation. All initial parameter guesses were automatically estimated. The background (offset from zero) was estimated to be the minimum intensity value of the profile, the amplitude of the profile was estimated to be the maximum intensity value minus the background, and the center position was estimated to be the position of the maximum intensity value. The FWHM of the Gaussian and Lorentzian functions, and the tubule diameter and PSF FWHM of the Lorentzian-convolved model functions were all estimated to be the FWHM of the profile, which was determined by counting the number of pixels with intensity values above the background plus half of the amplitude.

For NEP fitting, which was only applied using the Lorentzian-convolved model functions, the inner loop fitting was performed similarly to the standard fitting, with the only difference being that the inner loop did not try to optimize the PSF FWHM, and instead took this value as an input parameter parsed by the outer loop. The inner loop returned the mean of the mean squared errors (mean MSE) taken over each of the individual tubules fits, and this value was minimized in the outer loop fit using a Nelder-Mead minimization, where the only parameter directly controlled by the Nelder-Mead minimization was the PSF FWHM. In order to estimate the uncertainty of the PSF FWHM fit, the result from the Nelder-Mead minimization was passed as an initial guess to a Levenberg-Marquardt minimization to approximate the variance of the estimate. We used the Nelder-Mead algorithm for the primary parameter estimation because we found it to converge faster. In order to facilitate the estimation of the variance, the inner loop passed the Levenberg-Marquardt minimization an array of the residuals appended from each of the tubule profile fits. The variance estimate is calculated by multiplying the residual variance by the jacobian about the fit result.The standard deviation, however, is often below 1 nm for NEP fitting, which we take to be an underestimate of the uncertainty in the measurement.

Initial Guess Simulations

The simulations testing whether the fitting is robust to poor initial guesses used label-filled tubules, as the PSF width and tubule diameter are more highly coupled than for the surface-labeled tubules (see Fig. 3E, F, Fig. S7C, D, and Fig. S2D, E, I, J), suggesting that label-filled tubules would be more susceptible to poor initial guesses. The initial guesses for the fitting parameters were calculated as described above, however they were modified by a multiplicative factor we refer to as the 'guess multiplier'. The exception was the center position fit parameter, which was not affected by the guess multiplier, but instead was shifted from its initial calculation by one PSF FWHM (50 nm) for all simulated profiles. N=100 tubules of 115 nm diameter were simulated for each guess multiplier, using a PSF FWHM of 50 nm. Poisson noise was added to each profile, an example of which is shown in figure S3A.

Aberration Simulations

To simulate the effects of aberrations on NEP-fitting measurements, we simulated aberrated STED PSFs as follows. We used a vectorial, Fourier optics based, propagation strategy to generate 3D excitation, depletion, and detection PSFs from an arbitrary pupil function and took the in-focus slice as a 2D PSF which was convolved with a 2D projection of the target structures. PSFs were simulated using a 1.4 NA objective, with 650 nm excitation, 775 nm depletion, detection after a 1 Airy unit pinhole, and a steady-state exponential saturation model with a saturation factor of $I/I_{\text{sat}} = 25$. We assumed the depletion donut was slightly imperfect, and that the "zero" at the center was therefore 1% of the depletion profile maximum. This normalization was then

$$d_{\text{norm}}(x, y) = (1 - \delta) \frac{d(x, y) - d(0, 0)}{\max d(x, y) - d(0, 0)} + \delta, \tag{30}$$

where $(x, y) = (0, 0)$ is the center of the depletion profile $d(x, y)$ and we chose a δ of 0.01. The effective STED PSF was then calculated as

$$h(x, y) = h_{\text{excitation}}(x, y) h_{\text{detection}}(x, y) e^{-d_{\text{norm}}(x,y) I/I_{\text{sat}}}. \tag{31}$$

Aberrations were applied to the excitation and depletion PSFs, as well as the detection PSF (before being convolved with the pinhole). The aberrations were applied independently so each effective PSF corresponds to a single Zernike mode aberration of a given amplitude ranging from -1 rad to 1 rad. These PSFs were simulated over 4 µm x 4 µm areas with 5 nm pixel sizes. They fit nicely to a Gaussian profile, as expected for the steady-state continuous-wave assumption implicit in equation 31.

The fluorophore distribution of the label-filled and surface-labeled tubules were generated for 12 tubules evenly spaced in diameters from 50 nm to 160 nm. The thickness of the annulus for the surface-labeled tubules was set to be 4.5 nm to represent SNAP-tag labeling, and both surface-labeled and label-filled tubules were simulated with 5 nm pixel sizes over a 4 µm x 4 µm. For each aberrated PSF tested, these 12 tubules were convolved with the PSF, then a profile orthogonal to the long axis was extracted. These 12 profiles then constituted the ensemble of profiles for NEP-fitting for that PSF.

The aberrated PSFs were additionally fit separate of any tubule convolution in order to determine the ground-truth to compare the NEP-fitting results against. The 2D PSF was summed along the same dimension as the long-axis of the tubule to generate the 1D profile which was then fit to a 1D Gaussian. The sum was performed rather than a slicing operation in order to account for the two-dimensional convolution.

Cell Culture

COS-7 (ATCC, CRL-1651) cells were grown in a standard mammalian cell incubator with 5% CO_2 environment using phenol red free DMEM (Thermo Fisher Gibco) or DMEM/F-12 (Thermo Fisher Gibco) media supplemented with 10% FBS (Thermo Fisher Gibco). Cells were transfected by electroporation using a Super Electroporator NEPA21 Type II (Nepa Gene). Electroporation cuvettes with a 2 mm gap were loaded with 10^6 cells suspended in Opti-MEM (Thermo Fisher Gibco) and 2.5-10 µg DNA, depending on the desired expression level. Transfected cells were imaged 12-48 hours after electroporation.

Microtubule Samples

Microtubule samples were prepared using the method described by Huang et al (2016) (7). Briefly, COS-7 cells were grown on coverglass and pre-extracted using saponin before fixation with 3% paraformaldehyde + 0.1% glutaraldehyde. Mouse anti-α-tubulin antibody (Sigma-Aldrich, T5168) was used to label microtubules. A goat anti-Mouse antibody labeled with Atto647N (Sigma-Aldrich) was used as a secondary antibody. Samples were mounted in Prolong Diamond Antifade Mountant (Thermo Fisher Scientific) and imaged at room temperature.

ER Samples

The SNAP-Sec61β images used are a subset of images from a broader study on ER morphology (8). COS-7 cells were electroporated with either SNAP-Sec61β (9) or SNAP-KDEL (8) and plated in glass-bottom dishes (MatTek, 35 mm, no. 1.5). SNAP-tagged proteins were labeled immediately before imaging with 1 µM SNAP-Cell 647-SiR (New England Biolabs, S9102S) according to manufacturer's instructions. Living cells were imaged with 5% CO2, in Live Cell Imaging Solution (Thermo Fisher Scientific) supplemented with 15 mM D-glucose, and at 37 C using a stage incubator and objective heater.

STED Microscopy

Images were acquired using a Leica SP8 STED 3X equipped with a Onefive Katana-08HP pulsed laser as a depletion light source (775 nm wavelength) and a SuperK Extreme EXW-12 (NLT Photonics) pulsed white light laser as an excitation source. All images were acquired using a HC PL APO 100x 1.40 NA Oil CS2 objective. For living cells, imaging was performed with 8000 Hz line-scan speed and the cells were kept at 37 C with 5% CO_2. Fixed cells were imaged with a 1000 Hz line-scan speed, at room temperature. Images

were acquired using 16 line averages. All samples were imaged with 633 nm excitation and 775 nm depletion wavelengths, with depletion laser power of 110.6 mW for all images of ER. Emission light between 650-750 nm was collected using a detection gate set to 0.3-6 ns, on a HyD hybrid detector. The pinhole was set to 1 Airy unit.

Depletion Power Measurements

STED laser powers on the Leica SP8 STED 3X were measured using a microscope slide power meter sensor head (ThorLabs, S170C) with a digital handheld optical power meter console (ThorLabs, PM100D). Laser power at 775 nm depletion wavelength was measured using microscope settings to slowly scan a very small region with minimal beam blanking, which allowed us to detect the depletion laser light as a point rather than a scanned line. A 8192 x 8192 pixel region was scanned with 10 Hz line-scan speed, at minimum pixel size, and using bidirectional scanning at room temperature. These settings effectively scanned a 2.42 x 2.42 µm region with 295.68 pm sized pixels and 3.05 µs pixel dwell time. Laser power detected with these setting is equivalent to using the 'bleachpoint tool', as measured for an pulsed excitation laser (SuperK Extreme EXW-12, NKT Photonics) set at 660 nm wavelength. The depletion laser powers used in this work, 27.7 mW, 55.6 mW, and 110.6 mW, correspond to AOTF settings in the Leica software of 10%, 20%, and 40%, respectively.

Box Plots

The whiskers of all box plots represent the 10th and 90th percentiles of each distribution, the colored boxes cover the interquartile range, and the center line in each box denotes the median. Box plots and swarm plots were generated using the Seaborn Python package.

Software User Guide

Installation

NEP-fitting has flexible installation routes, but it is recommended to begin by installing the anaconda Python 2.7 distribution. Afterwards, the NEP-fitting plug-in and PYME can be installed by either using Anaconda Navigator (completely GUI-based) or by entering `conda config --add channels david_baddeley` followed by `conda install -c barentine nep-fitting` to the terminal. To install using Anaconda Navigator, open Navigator, add the channel by clicking the 'Add' button and entering 'barentine'. Do the same for the channel 'david_baddeley'. Now, click the 'environments' tab, search nep-fitting, select it, and click 'add'. Installation for these routes can be verified by running `STEDFitter` on the terminal/command prompt.

For contributing to or modifying the NEP-fitting plug-in, it is recommended to install using setuptools. Instructions for this are available at `github.com/bewersdorflab/nep-fitting`. If you install via setuptools, you will need to manually load the NEP-fitting plug-in after opening dh5view rather than simply calling `STEDFitter`; in this case, run `dh5view`, select an image to load, and select 'sted_psf_fitting' from the 'modules' drop-down menu.

Extracting Profiles

Extracting profiles begins by opening an image by `STEDFitter` in the terminal/command prompt. Click the square button with a diagonal line through it (1) to begin selecting line profiles. Click the starting point of the line, hold down, and release when the mouse is over the desired end-point. Press 'Add' (2). You should now see the line in the profile list at left. You can toggle the visibility of any line profile(s) by selecting it in the profile list, and clicking 'Show/Hide' (3). Similarly, one can remove a profile by selecting it in the list and clicking 'Delete' (4). The number of pixels to average the profile over (perpendicular to the line) can be set by typing a value into the 'Line width' box (5) and pressing enter (or clicking else-where). This updates the width for all profiles.

Once you have extracted the desired line profiles, you can save your profiles by clicking 'Save' (6). If you would like to analyze profiles from multiple images simultaneously, as is done in this work, you may append

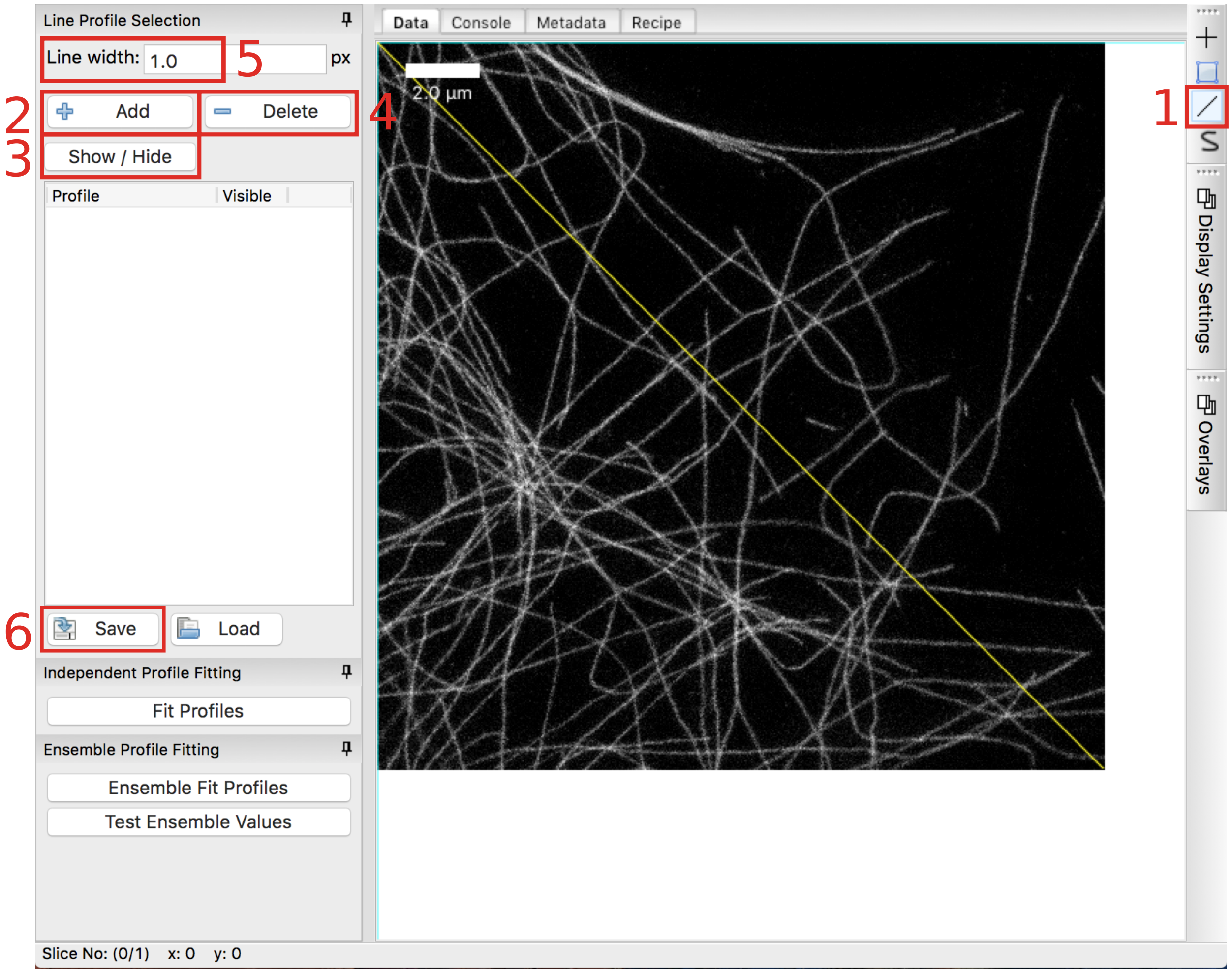

line profiles to the same file by clicking 'Save' and selecting the same file to save to. In this case, you will have to acknowledge a prompt which checks if you in fact would like to append to the previously written file.

Fitting Profiles

Line profiles can be extracted, as described above, or loaded using the 'Load' button (7) and selecting the HDF or JSON file containing the profiles in the resulting dialogue. After this is performed, standard, non-ensemble fits can be carried out by clicking 'Fit Profiles'(8), and NEP-fitting can be performed by clicking 'Ensemble Fit Profiles' (9). Each of these will open dialog boxes, asking which model to use during fitting, and potentially an initial guess for the PSF width. Upon completion of these fits, you will be prompted to save the fit results, after which a report will automatically be generated in HTML and opened as a new tab in your web browser.

The procedure for manually testing various PSF widths is quite similar to performing the fits, and is instead accomplished by clicking 'Test Ensemble Values' (10). You will be asked to enter the test values in a dialogue box, after which the values will be tested, and you will be prompted to save the report. After saving the report, it will again open as a new tab in your web browser.

Fitting, NEP-fitting, and ensemble testing can additionally be performed using the PYME bakeshop. The bakeshop can be opened by running `bakeshop`, and a recipe can be constructed either by adding recipe tiles manually, adding them using the 'Add Module' button, loading a saved recipe, or copying the text similar to the following (adjusted for the desired fit model, etc) into the box at right:

```
- nep_fits.EnsembleFitProfiles:
```

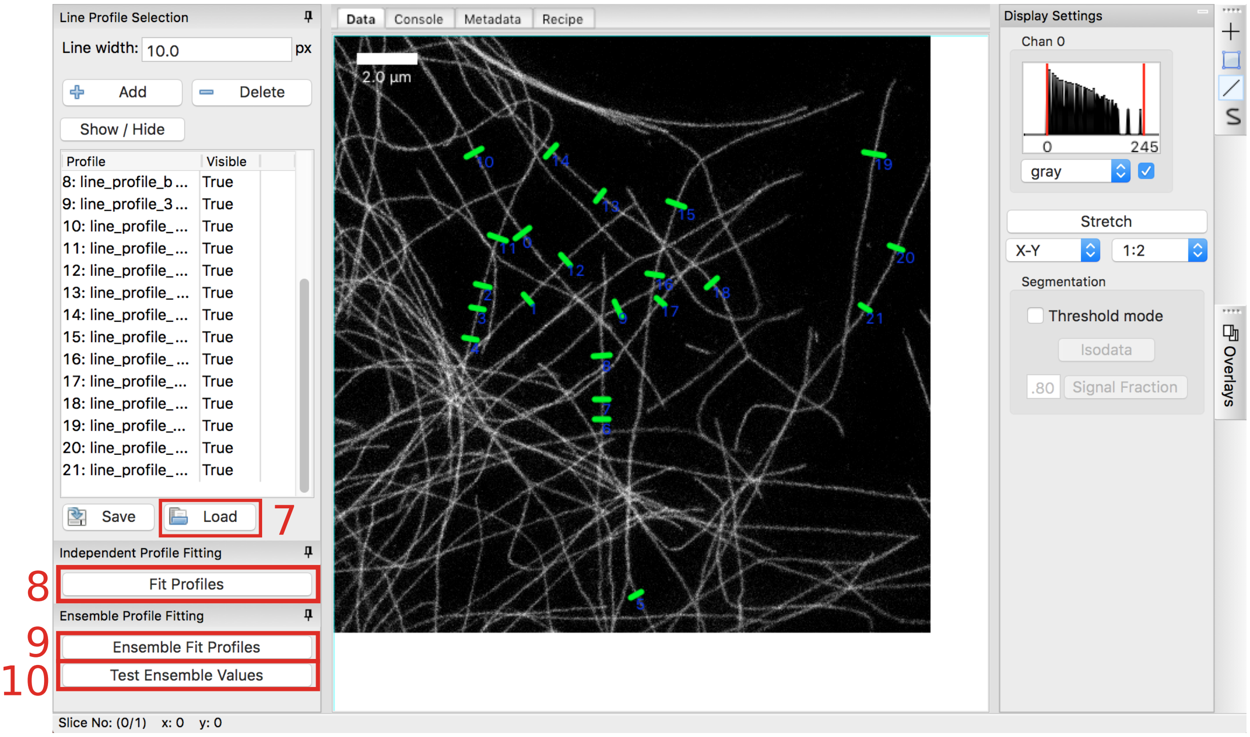


```
    ensemble_parameter_guess: 50.0
    fit_type: STEDTubule_Lumen
    hold_ensemble_parameter_constant: false
    inputName: line_profiles
    outputName: fit_results
- output.HDFOutput:
    filePattern: '{output_dir}/{file_stub}.hdf'
    inputVariables:
      fit_results: fitResults
    scheme: File
```

The input file(s) can be selected using filename patterns and clicking ‘Get Matches’. Finally, select the output folder by manual entry or through the ‘Browse’ dialogue, and click ‘Bake’.

References

[1] Vicidomini, G., A. Schönle, H. Ta, K. Y. Han, G. Moneron, C. Eggeling, and S. W. Hell, 2013. STED Nanoscopy with Time-Gated Detection: Theoretical and Experimental Aspects. *PLoS One* 8.

[2] Harke, B., J. Keller, C. K. Ullal, V. Westphal, A. Schönle, and S. W. Hell, 2008. Resolution scaling in STED microscopy. *Optics Express* 16:4154.

[3] Riley, K., M. Hobson, and S. Bence, 2006. Mathematical methods for physics and engineering. Cambridge University Press, third edition.

[4] Weisstein, E. W. Fourier Series–Semicircle. From MathWorld—A Wolfram Web Resource. `http://mathworld.wolfram.com/FourierSeriesSemicircle.html`.

[5] Weber, K., P. C. Rathke, and M. Osborn, 1978. Cytoplasmic microtubular images in glutaraldehyde-fixed tissue culture cells by electron microscopy and by immunofluorescence microscopy. *Proceedings of the National Academy of Sciences of the United States of America* 75:1820–4.

[6] Erickson, H. P., 2009. Size and shape of protein molecules at the nanometer level determined by sedimentation, gel filtration, and electron microscopy. *Biological Procedures Online* 11:32–51.

[7] Huang, F., G. Sirinakis, E. S. Allgeyer, L. K. Schroeder, W. C. Duim, E. B. Kromann, T. Phan, F. E. Rivera-Molina, J. R. Myers, I. Irnov, M. Lessard, Y. Zhang, M. A. Handel, C. Jacobs-Wagner, C. P. Lusk, J. E. Rothman, D. Toomre, M. J. Booth, and J. Bewersdorf, 2016. Ultra-High Resolution 3D Imaging of Whole Cells. *Cell* 166:1028–1040.

[8] Schroeder, L. K., A. E. S. Barentine, S. Schweighofer, D. Baddeley, J. Bewersdorf, and S. Bahmanyar, 2017. Nano-scale size holes in ER sheets provide an alternative to tubules for highly-curved membranes. *bioRxiv* .

[9] Bottanelli, F., E. B. Kromann, E. S. Allgeyer, R. S. Erdmann, S. Wood Baguley, G. Sirinakis, A. Schepartz, D. Baddeley, D. K. Toomre, J. E. Rothman, and J. Bewersdorf, 2016. Two-colour live-cell nanoscale imaging of intracellular targets. *Nature Communications* 7:10778.
